# A Causal Relation between Bioluminescence and Oxygen to Quantify the Cell Niche

**DOI:** 10.1371/journal.pone.0097572

**Published:** 2014-05-19

**Authors:** Dennis Lambrechts, Maarten Roeffaers, Karel Goossens, Johan Hofkens, Tom Van de Putte, Jan Schrooten, Hans Van Oosterwyck

**Affiliations:** 1 Department of Metallurgy and Materials Engineering, KU Leuven, Leuven, Belgium; 2 Prometheus, Division of Skeletal Tissue Engineering Leuven, KU Leuven, Leuven, Belgium; 3 Center for Surface Chemistry and Catalysis, KU Leuven, Leuven, Belgium; 4 Department of Chemistry and Biochemistry, The University of Texas at Austin, Austin, Texas, United States of America; 5 Molecular Imaging and Photonics, KU Leuven, Leuven, Belgium; 6 TiGenix NV, Leuven, Belgium; 7 Biomechanics Section, KU Leuven, Leuven, Belgium; CNR, Italy

## Abstract

Bioluminescence imaging assays have become a widely integrated technique to quantify effectiveness of cell-based therapies by monitoring fate and survival of transplanted cells. To date these assays are still largely qualitative and often erroneous due to the complexity and dynamics of local micro-environments (niches) in which the cells reside. Here, we report, using a combined experimental and computational approach, on oxygen that besides being a critical niche component responsible for cellular energy metabolism and cell-fate commitment, also serves a primary role in regulating bioluminescent light kinetics. We demonstrate the potential of an oxygen dependent Michaelis-Menten relation in quantifying intrinsic bioluminescence intensities by resolving cell-associated oxygen gradients from bioluminescent light that is emitted from three-dimensional (3D) cell-seeded hydrogels. Furthermore, the experimental and computational data indicate a strong causal relation of oxygen concentration with emitted bioluminescence intensities. Altogether our approach demonstrates the importance of oxygen to evolve towards quantitative bioluminescence and holds great potential for future microscale measurement of oxygen tension in an easily accessible manner.

## Introduction

In situ studies on the mechanisms of cell fate regulation in local microenvironments (niches) has gained considerable interest in the development of cell based therapies for disease and regeneration. These studies are very often complemented with bioluminescence imaging assays that yield valuable information on cell fate and behavior in a dynamic microenvironment. Likewise, specific niche components have been screened for their contribution to therapy outcome, including the matrix elasticity [1], presence of soluble and matrix-bound chemical agents in biomaterials [2], targeted and sustained release of cytokines from transplanted cells [3], and cell-adhesion mediated resistance against therapeutic agents [4].

Bioluminescence imaging relies on the activity of luciferase enzymes that act as catalyst for the conversion of luciferin to oxyluciferin, which is accompanied by the release of a photon [5]. Even though bioluminescence reporter imaging is a well-integrated technique for probing the biological function of living cells *in vitro* as well as in small-animal models [6], [7], the complexity of 3D cellular microenvironments precludes a quantitative interpretation of bioluminescent light [8]. Here we report on three major sources for the ambiguity in interpreting bioluminescence data obtained from cell-seeded hydrogels. At first the availability of bioluminescence substrate molecules (luciferins) to the luciferase enzymes which is dependent on active and/or passive transport from the site of application, secondly the accessibility to oxygen that is required for substrate oxidation, and finally the need for dynamic time point measurements of luciferase activity are all crucial determinants for making robust quantitative analyses. Although previous studies have elucidated the effects of oxygen concentration on the emitted bioluminescence intensity [9], [10], we show here how a mathematically validated model aids in resolving the oxygen dependent influences (changes in intensity, Michaelis-Menten kinetics, and decay rates) and how this model can be used to obtain quantitative measurements of the intrinsic bioluminescence intensity. Furthermore, we show that by careful analysis of the bioluminescence signal information can be obtained on local oxygen concentrations, evidencing a causal link between bioluminescence and oxygen.

## Results and Discussion

### Bioluminescence Intensities are Influenced by the Available Oxygen Concentration

**Figure 1:**
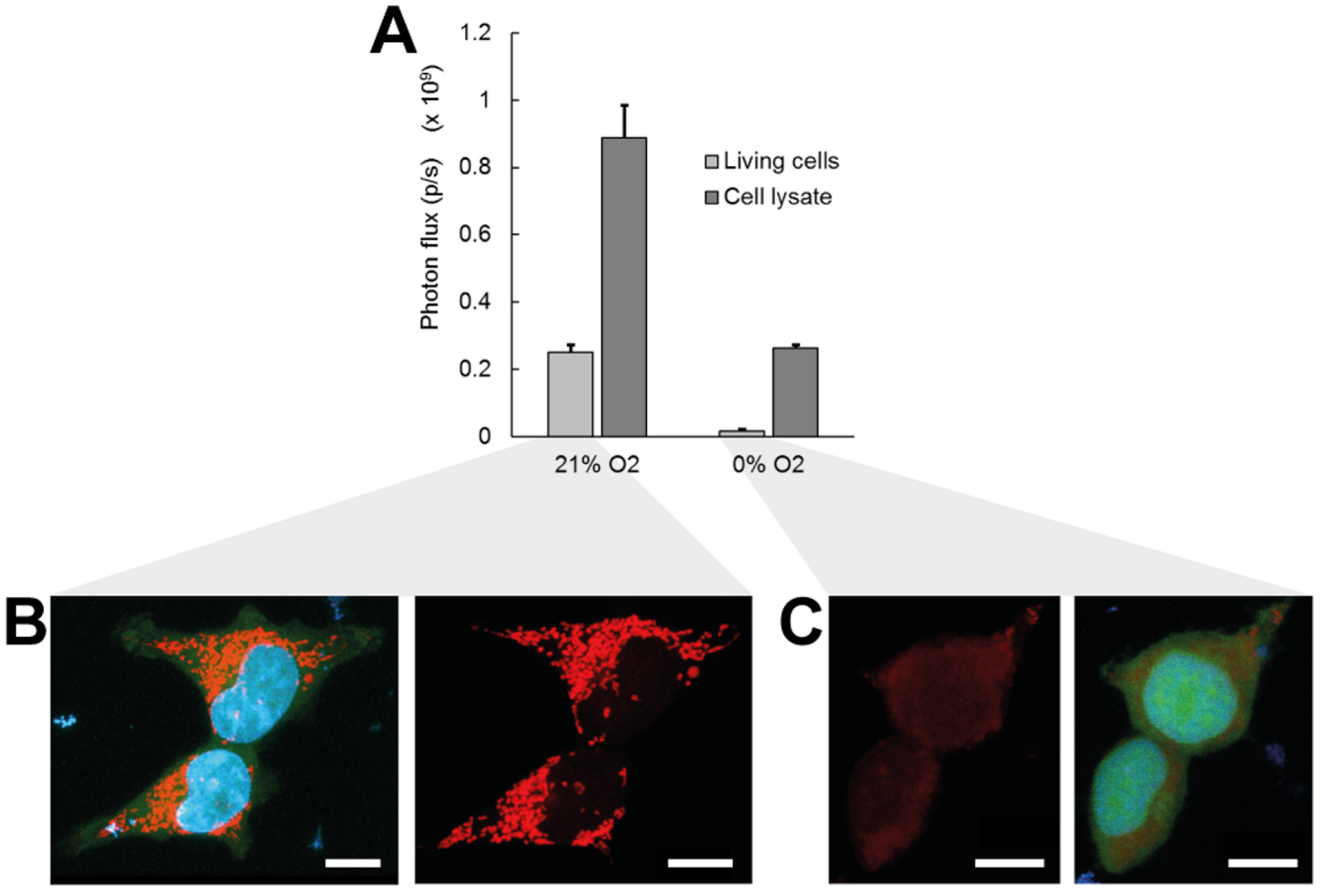
The activity of firefly luciferase in free solution or in intact 293T cells is dependent on available oxygen concentration. (**A**) Total photon flux at normoxic (21% O_2_) and hypoxic (0% O_2_, via addition of 1% Na_2_SO_3_) conditions emitted from a luciferase-dependent bioluminescence reaction in intact cells or from an equal concentration in cell lysates. Error bars, ±1 s.d. unit; *n* ≥5. (**B**, **C**) Confocal fluorescence imaging of cell mitochondria in cells exposed to (**B**) normoxic or (**C**) hypoxic oxygen concentrations. Images are maximum intensity projections of cell mitochondria stained with MitoTracker Red (red), cell nucleus stained with Hoechst (blue), and GFP signal (green) from stably transduced 293T cells. Left and right panels show the stained mitochondria with or without the other two channels, to reveal background fluorescence. Scale bar, 10 µm.

The importance of the oxygen availability for the bioluminescence reaction is best illustrated by the light flashing mechanism in the adult firefly *Photinus pyralis*. Tracheolar fluid levels in this system are acting as a diffusive barrier for oxygen supply and effectively control the light flashes emitted from photocytes in the firefly’s abdomen [11]. Oxygen-dependent luciferase activity is also observed *in vitro* in cell monolayer assays and has been used as reporter for cellular oxygen availability upon incubation with nitric oxide [12]. Here, we show that hypoxic conditions applied to firefly luciferase solutions result in a ∼3.4 fold difference in total photon flux as compared to normoxia (Fig. 1A). With the resolving power of currently available imaging equipment this fold difference should enable the quantification of oxygen-dependent luciferase activity. Conditions of normoxia (21% O_2_) and hypoxia (near 0% O_2_), representing the boundaries of oxygen availability in the artificial (atmospheric) and physiological (*in vivo*) cell environment [13], were applied in our experimental studies. Intermediate levels of oxygen concentration, which are difficult to maintain using regular gas exchange setups, were not directly applied but their influence was adapted from previously reported relations (which will be described below).

Other cofactors of the bioluminescence reaction (e.g. ATP) also account for a decrease in measured photon flux, but can often be directly or indirectly related to the influence of oxygen. Gradual accumulation of the inactive dehydroluciferyl-adenylate (L-AMP) complex within the cytoplasm of intact cells results in a lower photon flux compared to the free luciferase solution [14], [15]. The availability of free diffusing luciferin may be further decreased as membrane-bound ABC transporters not only control the net influx of luciferin into the cytoplasm but also require luciferin as a substrate for their activity [16]. In addition to the lower luciferase activity, ABC transporter-luciferin binding is also reflected in a slower apparent diffusivity of luciferin within cell-seeded hydrogels (Fig. S2). Comparison of photon fluxes from luciferase solutions relative to intact cells for corresponding luciferase concentrations yielded a difference of ∼3.5 fold under normoxic conditions, while a much larger difference of ∼16.5 fold was observed for the hypoxic environment (Fig. 1A). This effect mainly originates from a decrease in intracellular ATP content under hypoxia, concomitant with a reduction in mitochondrial membrane potential [10], [17]. We support this reasoning by visualization of mitochondria with a MitoTracker dye, clearly indicating a strong reduction of stained mitochondria in case of hypoxic incubation (Fig. 1B,C). Lower intracellular ATP concentrations potentially also influence the activity of ABC transporters, leading to a reduced influx of luciferin into the cytoplasm and hence a reduced photon flux [16].

### Reduced Oxygen Concentrations Induce Changes in the Bioluminescence Reaction Kinetics

**Figure 2:**
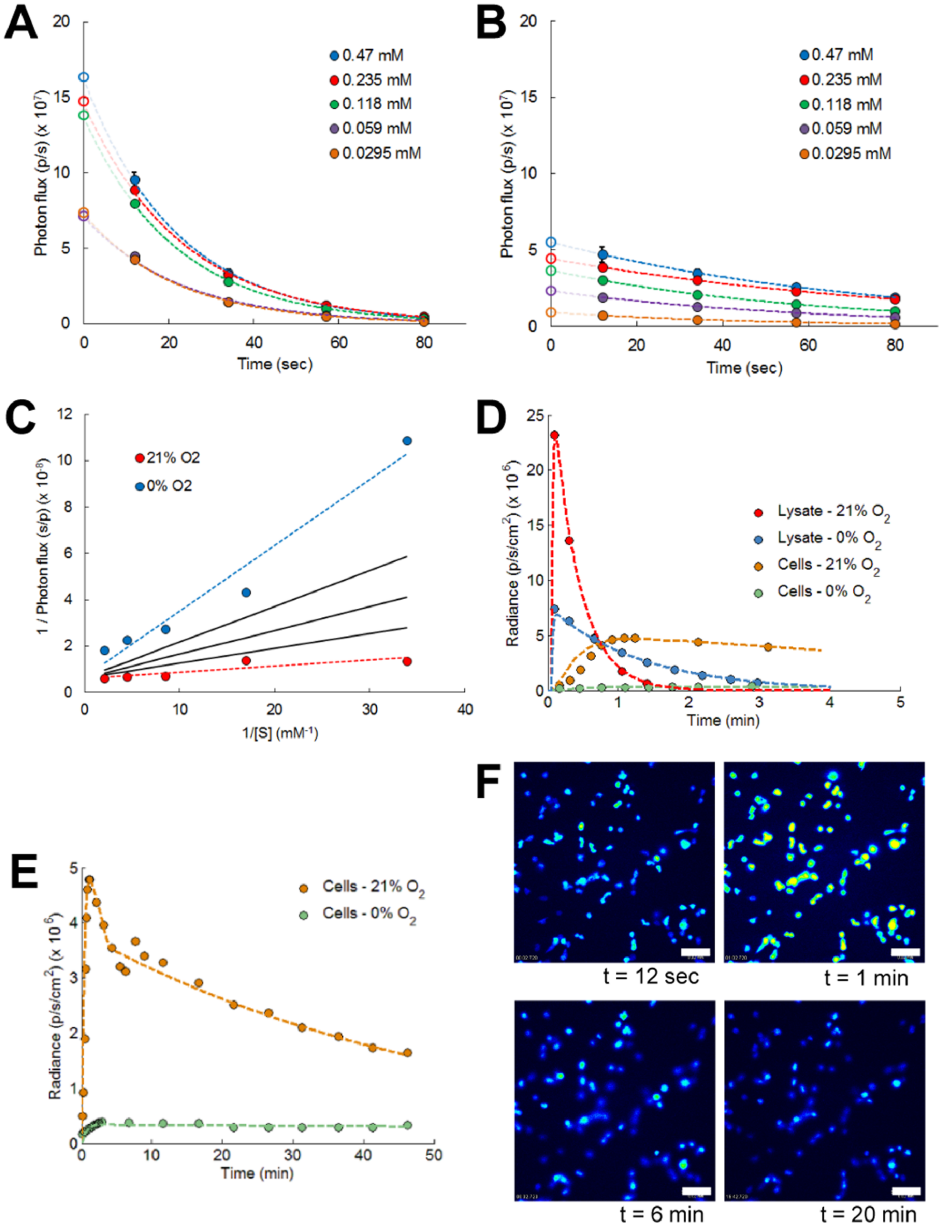
The available oxygen concentration induces changes in luciferase enzyme kinetics. (**A**, **B**) Dynamic time point analysis of luciferase activity with varying luciferin concentrations measured at (**A**) normoxic and (**B**) at hypoxic conditions. Measured data points were extrapolated with exponential functions to resolve the initial enzyme activity. Error bars, ±1 s.d. unit; *n* ≥3. (**C**) Lineweaver-Burk plots of initial luciferase activity show the influence of available oxygen concentration on bioluminescence kinetics-related parameters, at normoxia (*R^2^* = 0.74) and at hypoxia (*R^2^* = 0.94). Enzyme kinetics for intermediate oxygen levels (solid black lines –5, 10 and 15% O_2_ from top to bottom) are determined from a square root dependent relationship with available oxygen concentration. (**D**, **E**) Average photon flux emitted from cell lysates or intact cells at different oxygen concentrations. Simulation results (dashed lines) are shown for (**D**) short and (**E**) long term analyses. Cells incubated in hypoxic conditions display a delayed bioluminescence peak activity. (**F**) Bioluminescence microscopy of intact cells imaged at saturated (21% O_2_) oxygen concentrations (initial luciferin concentrations, 470 µM). Scale bar, 20 µm.

Oxygen dependent changes in initial bioluminescence reaction kinetics were determined from dynamic time point measurements of luciferase activity with varying luciferin concentrations. As peak intensities are reached within less than 1 second after reagent addition, fast operation and manipulation would be required to monitor initial light emission [18]. To circumvent these practical issues specifically for cell lysates, and avoid potential signal interference from measurement equipment, we extrapolated bioluminescent data that were obtained at later time points (Fig. 2A,B). Typical exponential decay of luciferase activity was observed under both normoxic and hypoxic conditions. Initial reaction velocities were subsequently transformed into Lineweaver-Burk plots in order to retrieve Michaelis-Menten kinetic parameters (Fig. 2C). Under normoxic conditions a higher substrate affinity was found as compared to hypoxia, with corresponding average Michaelis-Menten parameter values (*K_m,21_* and *K_m,0_*) of 42.6 and 938.4 µM respectively (Table S1). To estimate initial reaction velocities at intermediate oxygen tensions a square root dependency was adopted, similar to what has been reported for ATP-dependent kinetics [19]. This approach resulted in an oxygen dependent luciferase activity that matched with data from Moriyama *et al.* [10]. In this work, a previously published ordinary differential equation (ODE) model was extended to describe the average photon flux from cell lysates or intact human embryonic kidney 293T cells [20]. The extended model describes the kinetics of luciferin (extracellular as well as intracellular), firefly luciferase, and emitted photon flux, taking into account the oxygen dependency of photon generation (see Text S1 for further details). For intact cells we implemented an apparent Michaelis-Menten parameter (*K_m_*) which is 1.5 fold higher than in cell lysates. This value was obtained by model fitting and reflects the influence of a highly crowded cytoplasm on the luciferase activity [21], [22]. These results show great discrepancy with available literature data, where ∼7 fold changes in substrate affinity have been reported for cell lysates versus intact cells [23]. Most probably this literature data discrepancy can be attributed to the influence of luciferin membrane transport on the luciferase activity in living cells. In our analysis this influence was included explicitly, by introducing an additional transport term in the model equations (equation (1) and (2) in Text S1).

### Bioluminescence Decay Rates are Modified at Reduced Oxygen Concentrations

Intact cells show a significantly slower bioluminescence signal decay compared to the decay in luciferase solution, which is orchestrated by the concentration of cytoplasmic inorganic pyrophosphate (PPi) [14]. Also coenzyme A (CoA) concentrations can influence the decay dynamics. CoA stabilizes the photon flux by thiolysis of L-AMP into dehydroluciferyl-coenzyme A (L-CoA), which is a less powerful inhibitor than L-AMP on the bioluminescence reaction [24]. Under normoxic conditions the average exponential decay rates changed from 3948 s^−1^ for firefly luciferase extracted from cell lysates to 180 s^−1^ for intact cells. Hypoxic conditions have a remarkable influence on these decay rates with a decay rate of 1452 s^−1^ for cell lysates and 3 s^−1^ for intact cells (Fig. 2D). Cells exposed to an initial luciferin substrate concentration of 470 µM presented a bi-exponential decay in their luciferase activity (Fig. 2E,F) [23]. A fast initial decay (peak with decay rate of 180 s^−1^) which takes place in the first few minutes and which is indicative of a rapid substrate exhaustion, was followed by a slower decay rate of 27 s^−1^. Hypoxic environments repressed peak occurrence and transformed the diffusion-limited bioluminescence reaction into a more transition state-limited reaction. These changes were explicitly implemented into the model by a decrease in luciferin substrate affinity under hypoxia (*K_M_*, Table S1) and the introduction of a transition threshold parameter (*κ*, Text S1).

### Substrate Transport can be Monitored by Fluorescence Recovery after Photobleaching (FRAP)

**Figure 3:**
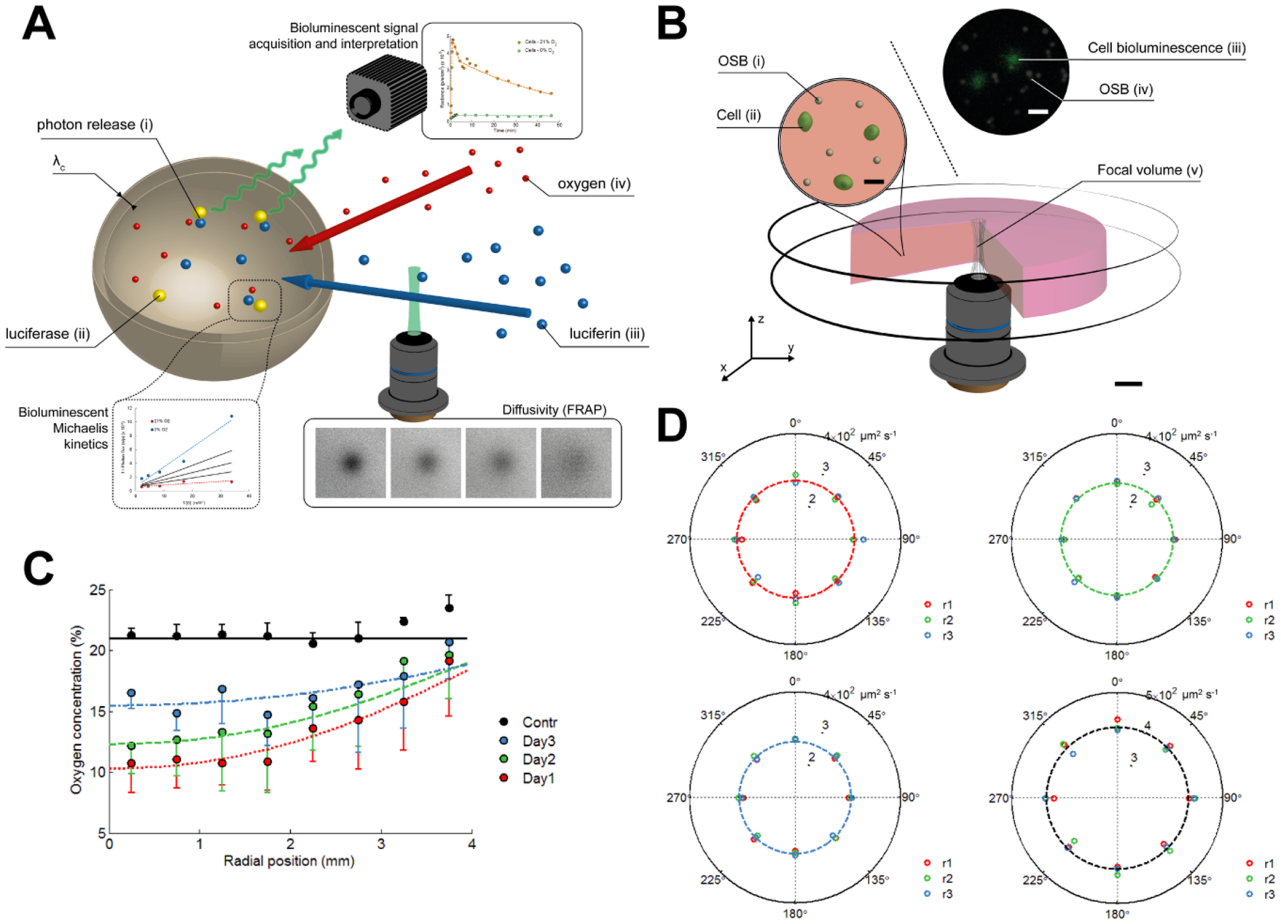
Overview of the mechanisms involved in bioluminescence photon emission from luciferase reporter cells embedded in a hydrogel. These mechanisms are implemented in the mathematical bioluminescence-oxygen model to decouple intrinsic bioluminescence intensities from the cellular oxygen environment. (A) Illustrated overview of the bioluminescence reaction in intact cells. Oxygen (iv) and luciferin (iii) pass through an extracellular matrix prior to cellular uptake. Diffusion rates are obtained from Fluorescence Recovery After Photobleaching (FRAP). Luciferin is actively transported across the cell membrane (thickness, *λ_c_*) and reacts with luciferase (ii) in the cell cytoplasm where this reaction is accompanied by the release of a photon (i). Oxygen availability in the cytoplasm modulates emitted light intensity and kinetics and is described by the Michaelis-Menten kinetics. (B) Setup for validation of the bioluminescence-oxygen model. Oxygen Sensing microBeads (i) and 293T cells (ii) are embedded in an agarose gel that is confined between 2 circular glass plates. Focal volume (v) imaged by combined bioluminescence and fluorescence microscopy reveals luciferase activity in single intact cells (iii) and local oxygen concentrations based on ratiometric intensities obtained from oxygen sensitive and insensitive dyes (iv). Scale bar, 1mm. Scale bar figure insets, 10 µm. (C) Radial oxygen concentration profiles in cell-seeded agarose gels measured by fluorescence intensities from embedded OSB. Colored lines indicate fitted oxygen profiles simulated by the oxygen model. Empty control gels (Contr) are imaged after 1, 2 and 3 days of incubation. Error bars, ±1 s.d. unit; *n* ≥3. (D) Polar plot of time-dependent changes in fluorescein tracer diffusion rates as measured by FRAP. R-axis of polar plot indicates tracer diffusion rate (µm^2^·s^−1^). FRAP analysis showed no significant difference in average values (dashed lines) at day 1 (red, top left panel), day 2 (green, top right panel), and day 3 (blue, bottom left panel). Spatial heterogeneities in diffusion rate within the agarose gel were determined from measurements at various radial (r1 = 1 mm, red; r2 = 2 mm, green; and r3 = 3 mm, blue) and angular positions in the gel. Empty control gel (black, bottom right panel) is shown as a reference. Measurements are performed in duplicate with a small shift in spatial position, *n* ≥3.

As indicated before, active cellular membrane transport is not the sole mechanism which controls the availability of luciferin to the cytoplasmic luciferase. When cells are embedded in an extracellular matrix (ECM), luciferin molecules will be transported through this matrix prior to intracellular uptake (Fig. 3A). This necessitated the introduction of diffusion in the model and therefore resulted in a system of partial differential equations (PDEs). Using fluorescence recovery after photobleaching (FRAP) [25], we were able to obtain quantitative measures of substrate diffusion rates at different positions in space and time, in order to rule out any changes in diffusivity. Measurements were performed with an optimized concentration of fluorescein tracer (see Fig. S2). FRAP-based ratios of tracer diffusivity in culture medium versus agarose hydrogel were validated by comparing diffusion rates obtained from Fluorescence Correlation Spectroscopy (FCS) (Fig. S3).

### Oxygen independent Bioluminescence Intensities are a Good Measurement of the Active Bioluminescent Cell Population

Oxygen- and transport-related parameters influencing luciferase activity were subsequently implemented into the PDE-based model. Time-dependent luciferase activity showed a typical exponential decay which was described mathematically by a first order chemical kinetic equation (Equation (3) in Text S1) [26]. Substrate diffusion through cell membranes also accounted for the observed time-dependency of bioluminescent reaction rates, which by virtue of the induced spatial heterogeneities resembled fractal-like reaction kinetics [27]. The afore-mentioned model fits the experimental data points with good accuracy, thereby enabling reliable quantitative insights into the bioluminescence reaction kinetics for both cell lysates and intact cells (Fig. 2D,E). The influence of spatial organization on the emitted bioluminescence from luciferase reporter cells was validated for a homogeneously cell-seeded (∼1×10^6^ cells·ml^−1^) cylindrical agarose hydrogel (Fig. 3B). Circular glass plates confined the hydrogel in axial direction, preventing axial diffusion of oxygen and luciferin. This resulted in an axisymmetric setup which simplified further analysis.

Microscale measurements of oxygen concentration were performed by integration of fluorescent oxygen sensitive microbeads that we previously developed [28] (Fig. S4). Stable read-outs of oxygen concentration over a time period of 3 days were obtained for control gels in which no cells were embedded (Fig. S4F). Radial oxygen concentration profiles from cell-seeded hydrogels displayed a gradual decrease towards the center with an averaged availability of oxygen that increased over time (Fig. 3C). Diffusivity measurements obtained from FRAP experiments in cell-seeded hydrogels displayed no significant changes in tracer diffusion rate, with an average value of ∼350 µm^2^s^−1^ (Fig. 3D). As nearly constant values in space and time for the tracer diffusion rate were measured it can be concluded that the time-dependent increase in radial oxygen concentration profiles was induced by a decrease in initial oxygen consumption rate (OCR) by the cells with time (Fig. S4G). 293T cells are able to respond to reduced oxygen availability by altering cytochrome c oxidase (COX) subunit composition via a hypoxia-inducible factor 1 (HIF-1) -dependent mechanism [17], that results in a decrease of their OCR. We implemented these time-dependent changes in OCR directly into the model to accurately match the measured radial oxygen profiles (Fig. 3C and equation (4) in Text S1).

**Figure 4:**
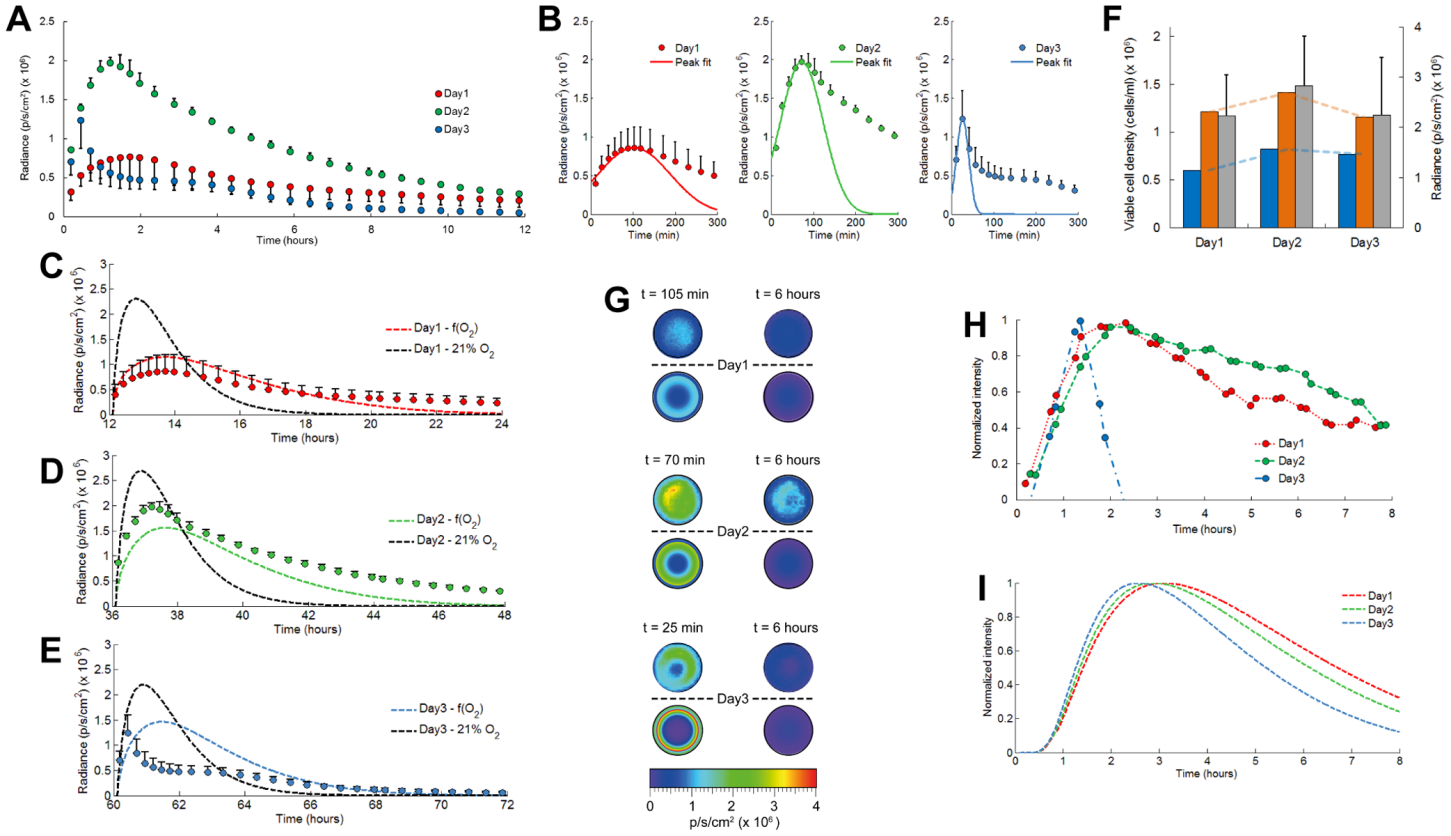
Validation of the bioluminescence-oxygen model for quantitative interpretation and analysis of bioluminescent light emitted from cell-seeded hydrogels. (**A**) Average photon flux measured for luciferase reporter 293T cells embedded in agarose gels that are axially confined by circular glass plates. Dynamic time point measurements were performed during 12 hour periods. (initial luciferin concentration, 47 µM) Error bars, ±1 s.d. unit; *n* ≥3 (**B**) Emission peak intensities were fitted by Gaussian functions. Time-lapse measurements of the average emitted photon flux were compared with simulation results from the bioluminescence-oxygen model in presence or absence of oxygen gradients at day 1 (**C**), day 2 (**D**), and day 3 (**E**). (**F**) Comparison of simulated peak emission intensities in presence (blue) or absence (orange) of oxygen gradients, with average viable cell densities obtained from quantitative DNA analyses (gray). Error bars, ±1 s.d. unit; *n* = 6. (**G**) 2D bioluminescence profiles of cell-seeded agarose gels imaged from top position (hydrogel diameter, 8 mm). Profiles above the dashed lines are measured with the IVIS 100, and profiles below are simulated from the bioluminescence-oxygen model. The left column shows activity at the peak position and the right column shows activity after 6 h (steady-state condition) (**H**) Bioluminescence microscopy of 293T cells embedded in agarose at the central hydrogel position and (**I**) comparison with the simulated activity in the hydrogel center.

Dynamic time point measurements of the average photon flux emitted from the cell-seeded hydrogels revealed the presence of a fast bioluminescence intensity peak that fades away slowly with a decaying signal that was detectable for several hours (Fig. 4A). Peak intensities reached maximum values after 2 days of cultivation and decreased gently with longer cultivation time (Fig. 4B). These results did however not entirely correlate to the quantitative DNA and viability measurements, representing the active bioluminescent cell population and clearly being oxygen-independent readouts (Fig. S5). To decouple bioluminescence signal reshaping caused by oxygen availability from the spatial distribution of luciferase activity, we removed cell respiration in our model, hence maintaining a saturated (and uniform, 21%) oxygen level within the hydrogel and therefore oxygen-independent bioluminescence signal intensities. A prominent role for oxygen in reshaping the bioluminescence signal was clearly observed (Fig. 4C–E). When saturated oxygen conditions were simulated the obtained peak signal intensities matched closely the cell activity measurements (Fig. 4F). Radial profiles of the bioluminescence signal emitted from encapsulated 293T cells indicated high activity near the hydrogel edge at early time points that gradually leveled off towards the center after longer measurement times (Fig. 4G, Video S1). With increasing cultivation times the observed effect of high edge activity became more pronounced and was in good agreement with our simulation results. Combined, these data show a strong influence of oxygen availability on the activity of luciferase reporter cells which should be taken into account when intrinsic (i.e. independent of other environmental factors) measurements and error-free interpretations of bioluminescence intensity are pursued.

### Oxygen Concentrations within Cell-seeded Hydrogels can be Derived from Bioluminescence Signal Analysis

With longer cultivation times peak bioluminescence also occurred faster after luciferin substrate addition, in conjunction with a reduced full width at half maximum (FWHM) and a faster decay rate of the bioluminescence signal (Fig. 4B and Fig. S6A–C). Bioluminescence microscopy of cell activity at the hydrogel center revealed comparable parameter trends, though the change in signal decay rate with culture time was more pronounced (Fig. 4H,I and Fig. S6E–G). Strong discrepancy with the simulated decay rate was observed at 3 days of cultivation. This difference could therefore indicate that a luciferin concentration dependent decay would be required to accurately fit the experimental data, which is supported by a strongly reduced substrate availability in the hydrogel center (Fig. S7) and maintenance of a concentration dependent substrate gradient (partitioning) across the cell membrane (Fig. S8) [14]. A good correspondence in the evolution of bioluminescence peak parameters and available oxygen concentration was clearly observed, evidencing the causal link (Fig. S6D,H). These results hence show great promise towards quantifying averaged or localized oxygen concentrations in cell-based systems that are based on univocal relations between bioluminescence and oxygen.

Oxygen is a critical component of the cell microenvironment, such as for the determination of cell fate in stem cell niches [29], [30]. As such, new non-invasive technologies and methodologies should be developed that are capable of oxygen measurement and control in 3D environments in space and time. In this respect, the methodology as stated above holds great potential as a tool for easily-accessible measurements of oxygen concentration directly from the time-dependent bioluminescence signal emitted within a dynamic microenvironment. Further validation is required to assess practical use of the identified bioluminescence peak parameters in determining oxygen for other experimental setups.

## Materials and Methods

### 293T Cell Culture and Transduction

Human embryonic kidney 293T cells were maintained in Dulbecco’s modified Eagle’s medium (DMEM) with Glutamax (Invitrogen, Merelbeke, Belgium) supplemented with 10% irradiated fetal bovine serum (Gibco), and 1% antibiotic-antimycotic (A/A) solution (100 units·ml^−1^ penicillin, 100 µg/ml streptomycin, and 0.25 µg/ml amphotericin B; Invitrogen). Cells were cultured at 37°C in a humidified atmosphere containing 5% CO_2_. Medium was refreshed every 2–3 days and cells passaged when sub-confluent.

293T cells were transduced with a lentiviral vector (pCH-EF1a-3flag-fLuc-T2A-eGFP-Ires-Bsd, 3.1×10^8^ TU·ml^−1^) which was a kind donation from Dr. Greetje Vande Velde (MoSAIC, KU Leuven). The day before transduction, cells were seeded in a 96-well plate at 1×10^4^ cells per well. On the day of transduction, medium was replaced by DMEM containing serial dilutions of the vector and incubated for 24 hours. After 24 hours, medium was replaced with DMEM containing 1 µg·ml^−1^ blastidicin for antibiotic selection of the stably transduced cell population, and was continued for 2–3 weeks. Transduction efficiencies were analyzed by flow cytometric analysis (FACS). Luciferase expression levels of the transduced cells were stable over time (Fig. S1).

### In vitro Luciferase Activity Assay

Stable read-outs of luciferase activity were obtained by thoroughly mixing 5 µl solutions of recombinant luciferase (QuantiLum, Promega), firefly luciferase extracted from 293T cells or intact luciferase-reporting 293T cells in suspension in 45 µl of Luciferase Assay Reagent (Promega). Incubation resulted in a glow-type reaction that lasted longer than ∼1 min and total photon fluxes were measured using black-walled 96 well plates in the IVIS 100 imaging system (PerkinElmer) with 1 s acquisition time. Image processing and analysis were performed in IGOR Pro software (WaveMetrics). Final concentrations of recombinant luciferase solutions were obtained upon dilution of stock solutions with purified water from a Milli-Q system (Millipore) (Fig. S1). Measurements on cell lysates were performed by lysing 293T cells in Cell Culture Lysis Reagent (Promega) with a final concentration of 1×10^5^ cells·ml^−1^ and were compared to an equal concentration of 293T cells in DMEM (Fig. S1). Hypoxic conditions were induced by addition and incubation (>15 min) of 1% Na_2_SO_3_ in luciferase solution and assay reagent before mixing. All bioluminescence measurements were performed at 37°C incubation temperature.

### Dynamic Time Point Analysis of Free Luciferase Activity

Flash-type bioluminescence reactions were provoked by mixing 10 µl solutions of recombinant luciferase (0.1 nM) with 100 µl luciferin solutions (Beetle luciferin potassium salt (Promega) in 10 mM tricine, 1.07 mM magnesium carbonate pentahydrate, 2.67 mM magnesium sulfate, 0.1 mM ethylenediaminetetraacetic acid (EDTA), 0.5 mM ATP sodium salt, 0.27 mM coenzyme A sodium salt and 33.3 mM dithiothreitol (DTT)). Initial reaction velocities were obtained by fitting exponential decay functions through the measured data points and by extrapolation of the luciferase activity to the initial time point. The initial time point was found by application of a ratiometric criterion between total emitted photon fluxes at normoxia and hypoxia, that we measured via a stable glow-type reaction. This ratio indicated ∼3.37 fold difference in initial reaction velocities. Photon fluxes were measured using black-walled 96 well plates in the IVIS 100 imaging system (PerkinElmer) with 1 s acquisition time.

### Dynamic Time Point Analysis of Luciferase Activity in Intact Cells

Cell densities (1.09×10^5^ cells·ml^−1^) used for dynamic time point analysis were calculated from the average intracellular luciferase concentration (0.92 amol·cell^−1^) as the equivalence of a 0.1 nM free recombinant luciferase solution. Cells were suspended in DMEM and at ∼20 min before signal measurement, and cells used for the hypoxic condition were resuspended in DMEM containing 1% Na_2_SO_3_. Longer hypoxic incubation times resulted in a modest decrease in luciferase activity. Luciferase activity was measured after mixing 10 µl of cell suspension with 100 µl of luciferin solution (Beetle luciferin potassium salt in DMEM). Photon fluxes were measured using black-walled 96 well plates in the IVIS 100 imaging system (PerkinElmer) with 1 s acquisition time. The stability of luciferase expression levels for stably transduced cells that are exposed to different oxygen concentrations has been shown elsewhere [10].

### Single-cell Bioluminescence Microscopy

Glass coverslips were coated with 500 µl Poly-L-Lysine (PLL, Sigma) solution (0.1%, 5 min), rinsed with Milli-Q water and dried overnight. We plated 1×10^5^ 293T cells on PLL-coated coverslips. After overnight cell attachment these plates were placed on the stage of a luminescence microscope (LuminoView 200, Olympus). Cells were incubated with a 470 µM luciferin solution at 37°C (Solent Scientific). Bioluminescence was imaged with a UPLSAPO 60× water objective (NA: 1.2) and transmitted to a cooled CCD camera (ImagEM512, Hamamatsu Photonics) mounted on the bottom port of the microscope. Time-lapse images were collected with 5 s acquisition times and EM gain was set at 1200× (photon imaging mode, Hamamatsu) (Fig. 2F).

### Fluorescence Recovery after Photobleaching (FRAP) Analysis of Self-diffusivity

Low melting point agarose gels (Invitrogen) with a final concentration of 2% were prepared on glass coverslips (thickness, 2 mm and diameter, 8 mm). Gels were incubated with DMEM containing various concentrations of fluorescein (Sigma). After overnight incubation slides and gels were transferred to the stage of a confocal fluorescence microscope (FluoView 1000, Olympus) equipped with a UPLSAPO 10× air objective (NA: 0.40) used for observation. Measurements were performed at 37°C. Fluorescence images were collected with the systems PMT. A 488 nm Ar laser was used to bleach and also monitor the recovery of fluorescein tracer molecules. The pinhole size was set to 50 µm. Images were acquired with 72 ms intervals during a 3 s scanning period, and consisted of 256×256 pixels with a pixel size of 0.497×0.497 µm (zoom factor: 10×). Prebleaching images were acquired to compensate for non-uniform light illumination. Circular regions (radius, 10 µm) were bleached (total bleaching time, 13 ms) with the laser in tornado-scan mode (SIM Scanner, Olympus) to obtain centered, fast, and uniformly bleached spots with an approximate Gaussian shape. Image analysis was performed with a program written in MATLAB (The MathWorks, Natick, MA). The method implemented into this program is based on a spatial frequency analysis of circularly averaged radial intensities of each image [25]. In brief, the recovery of fluorescent tracer was modeled according to Fick’s second law. An analytical solution to this equation was obtained via the Hankel transform. Circular averaging on the radial intensities was performed to reduce the noise in the intensity profiles. Finally the analytical solution was fitted to the experimental curves using a nonlinear curve fitting algorithm in MATLAB. We also verified the independence of diffusion rates on spatial frequency, as our setup is characterized by Brownian diffusion. Analysis was performed for a single diffusing component with the fraction of immobile molecules set to zero. The fluorescein tracer concentration was optimized to detect changes in fluorescein diffusivity, caused by local matrix degradation, with maximal sensitivity (Fig. S2B). A fluorescein concentration of 25 µM was found to be optimal. Spatial heterogeneities in tracer diffusivity were detected by FRAP imaging at different circumferential positions along 3 radial positions in the gel (imaging depth, 150 µm). Measurements were performed in duplicate on each gel with a small shift in spatial position.

### Fluorescence Correlation Spectroscopy (FCS)

Agarose gels (2% in DMEM supplemented with various concentrations of Rhodamine B) were prepared between two glass coverslips separated by ∼450 µm thick spacers. FCS measurements were performed on a custom-built fluorescence spectro/microscopy setup using a 532 nm laser line (Spectra-Physics CW DPSS Excelsior laser; the maximal laser power of 500 mW was attenuated by means of neutral density filters (New Focus)) for fluorophore excitation. The excitation light was circularly polarized by means of a λ/4 waveplate (Thorlabs), expanded using a telescope arrangement (to a collimated beam of about 7 mm diameter (1/*e* ^2^ intensity), and directed via a dichroic beamsplitter (z532rdc, Chroma Technology, Rochingham, USA) into the oil-immersion objective (UPLSAPO 100×, NA = 1.4) of an inverted optical microscope (Olympus IX71). The center of the confocal volume was positioned 5 µm above the coverslip surface. Fluorescence light was collected by the same objective and was guided, after passing through the dichroic, through a 50 µm pinhole (Linos/Qioptic) in order to reject light from out-of-focus regions. The emitted light was filtered by a longpass filter (HQ545LP, Chroma Technology, Rockingham, USA) focused on a τ-SPAD photon counting module (PicoQuant, active area 150 µm in diameter), connected to a HydraHarp 400 TC-SPC module. Measurements were performed until at least 3 million photon-detection events had been recorded, typically requiring a few minutes. Calibration of the one-focus FCS setup was performed using a solution of Rhodamine 6G in Milli-Q water (D ≈ 372 µm^2^ s^−1^) [31] using the same excitation laser power as for the samples under investigation (1.69 kW cm^−2^ at the sample). Temporal autocorrelation curves were calculated from the measured fluorescence intensity fluctuations (photon count time traces) within the SymPhoTime software (PicoQuant). These autocorrelation curves were fitted in IGOR Pro (WaveMetrics) to an expression for autocorrelation decay due to anomalous diffusion [32],
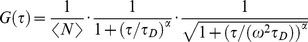
(1)


In this expression, 〈*N*〉 is the average number of fluorescent dye molecules in the confocal volume element, 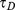 is the diffusive time of dye molecules, *α* is the degree of anomalous subdiffusion, and *ω* considers the extension of the confocal volume along the optical axis ( = 〈*w*_z_〉*/*〈*w*_xy_〉). The diffusive time is related to the translational diffusion coefficient *D* as 
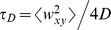
 for *α* = 1. Anomalous subdiffusion can occur due to macromolecular crowding [32]. As fluorescent tracer molecules applied in this study are small, we did not expect strong deviations in the anomality parameter, which is supported by Figure S3. All FCS measurements were performed at RT (21°C).

### Time-lapse Diffusion Measurements

Intrinsic diffusion rates were measured with an optically transparent diffusion setup in which fluorescent tracer movement was imaged using a confocal fluorescence microscope (FV1000, Olympus). Beetle luciferin (MW: 318 g·mol^−1^, Promega) was used as a tracer molecule. Luciferin is fluorescent with an emission maximum at 537 nm and absorption maximum near 328 nm in acidic solutions and near 384 nm in basic solutions [33]. The emission maximum of the oxyluciferin complex is 523 nm [33], [34]. Tracers were excited with a 375 nm laser line. Imaging was performed with a DM375-405/515/635 primary beam splitter in combination with a BA535-565 emission filter for visualization of luciferin diffusion and a BA505-540 emission filter for detection of accumulated oxyluciferin complex. Tracer motion was dependent on a concentration gradient induced between a saturated tracer concentration (100 µM) in DMEM and a tracer-free 2% agarose gel. Agarose gels were produced in glass Pasteur pipette tips (ID, 1.05 mm; OD, 1.7 mm) and at the onset of the diffusion experiment were connected to transparent silicon tubing (ID, 1.57 mm; Cole-Parmer) containing the saturated tracer solution. Image sequences were acquired with 5 min intervals during a 3 hour scanning period. An area of 1920×640 pixels was visualized with a pixel size of 1.42×1.42 µm. Imaging was performed with a UPLSAPO 10× air objective (NA: 0.40), focused on the middle plane of the agarose gel, and with a pinhole size of 400 µm. Measurements were performed at 37°C. Empty agarose gels were imaged to correct for background intensities. Intensity profiles acquired during the tracer diffusion experiment were normalized to the average intensity measurements obtained from a tracer-saturated agarose gel. Image sequences were processed in Fiji (NIH, Bethesda, MD, USA). Luciferin diffusion rates were obtained by least squares fitting an analytical solution of Fick’s second diffusion law (diffusion in semi-infinite media [35]) to the resulting averaged axial intensity profiles,
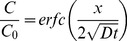
(2)Where, *C* is the concentration of tracer diffusing into the agarose gel, *C_0_* is the initial tracer concentration in the tracer-saturated agarose gel, and *D* is the diffusion coefficient obtained by profile fitting. The profile fitting algorithm was implemented in MATLAB. The initial saturated tracer concentration (in DMEM) contained in the silicon tubing was assumed to remain constant during the diffusion experiment. Validity of this assumption was verified by comparing the analytical solution of luciferin diffusion with a numerical solution of the diffusion problem implemented in COMSOL (COMSOL Multiphysics, Burlington, MA, USA).

### OSB Production and Calibration

OSB are microscale oxygen sensors that consist of a core material, on which an oxygen sensitive and an oxygen insensitive dye are deposited, and a shell material, that prevents the dyes from leaching and protects the cells against potential dye toxicity. The protocol for OSB production is based on a strategy we previously developed to produce avidin-coated OSB [28] and is composed of two steps.

The core material of the OSB consists of silica gel (spherical silica gel, 45809, Alfa Aesar) and has a diameter of 5 µm. An initial amount of 50 mg silica gel spheres were brought into suspension with a 1 ml aqueous NaOH (0.01 N) solution and were mechanically stirred at 250 rpm for 30 min. Oxygen measurements relied on the incorporation of two fluorescent dyes; an oxygen sensitive Tris (4,7-diphenyl-1,10-phenanthroline) ruthenium (II) dichloride (76886, Sigma) and an oxygen insensitive reference fluorophore, Rhodamine 6G (56226, Fluka). Two 250 µl solutions were prepared containing these molecules in concentrations of 500 and 50 µM, respectively. These solutions were poured into the silica gel solutions and mechanically stirred (1 hour, 250 rpm). Afterwards the beads were diluted with 4 ml Milli-Q water and washed and centrifuged (1300×g, 20 min) three times with Milli-Q water and once in ethanol. Beads were resuspended in 6.5 ml ethanol. Coating of silica gel spheres was performed via a Stöber seed growth method of silica shells described by the van Blaaderen group [36]. In brief, a solution of ammonia in water (25 w/v % NH_4_OH in Milli-Q water, 250 µl) and tetraethyl orthosilicate (99% TEOS, 32.5 µl, 86578, Sigma) was added to the silica spheres suspended in ethanol. Next this solution was mechanically stirred (250 rpm) for 18 hours. Suspended beads were collected by centrifugation (1300×g, 20 min) and washed three times in PBS solution (5 ml). The bead solution was autoclaved and supernatant removed after centrifugation (1300×g, 10 min). Beads were incubated at low temperature (4°C) in 2 ml DMEM and stored in the dark at a density of ∼3×10^8^ beads·ml^−1^. The amount TEOS solution to be added to the Stöber seed growth reaction, was determined from the relation [37], [38]:
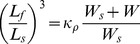
(3)where *L_s_* is the initial size of particles before shell growth, *L_f_* is the final size after shell growth (to obtain ∼400 nm silica shell thickness), *W_s_* is the weight of SiO_2_ initially present as seeds, *W* is the amount of SiO_2_ added to the reaction mixture in the form of TEOS, and *κ_ρ_* = (*ρ_0_*/*ρ*)^1/3^ is a correction factor accounting for the differences in density between seed and final particles [39], with *ρ_0_* the density of the initial particle and *ρ* the density of the final particle.

OSB were calibrated by exposure to hypoxic (0% O_2_ via mixing with 1% Na_2_SO_3_) and normoxic (21% O_2_) oxygen conditions in DMEM. Microbeads were imaged on an inverted confocal laser scanning microscope (FV1000, Olympus) with a UPLSAPO 20× air objective (NA: 0.75). Excitation of the fluorescent beads was performed with a 488 nm Ar laser. Fluorescence emission was detected with a DM405/488 primary beam splitter in combination with a BA505-540 emission filter for detection of the Rhodamine 6G and a BA575-675 emission filter for detection of the ruthenium complex. Image stacks were acquired along the radial direction and consisted of 640×640 pixels with a pixel size of 0.165×0.165 µm and images (n = 5) acquired in z-direction were separated by a distance of 50 µm. Experiments were performed at 37°C incubation temperature (Solent Scientific).

Regions of interest (ROI) were defined around individual beads for calibration and measurement of OSB. Images were processed in Fiji with a threshold macro. Average signal intensities were subsequently inserted into the Stern-Volmer equation. Further details on the calibration procedure can be found elsewhere [28].

### Focused Ion Beam Milling and Scanning Electron Microscopy

OSB were placed on SEM specimen holders and coated with a thin conducting platinum layer by a sputter coater system (Quorum Q150 TS, Quorum Technologies Ltd, Laughton, UK). FIB ablation and SEM imaging were performed on a Nova 600 Nanolab dual-beam system (DualBeam™-SEM/FIB, FEI, Hillsboro, USA). Beams are separated from each other by an angle of 52°. Therefore, vertical cutting of the OSB with the FIB proceeded by tilting the stage with this angle and vertical cross-sections with the electron beam were also observed at this angular position. The SEM images were generated in high vacuum mode using an acceleration voltage of 5 keV and an Everhart Thornley detector.

### Assembly of Setup for Validation of PDE-based Model

Low melting point agarose gels (2% in DMEM, Invitrogen) containing 1×10^6^ cells·ml^−1^ and supplemented with 2.5×10^6^ OSB·ml^−1^, were prepared between two glass coverslips. The slides were separated by a 2 mm thick Teflon spacer, which consisted of two parts and at the central position contained a cylindrical hole (diameter, 8 mm) that served as a mold for the gel production. After the gel was poured into this hole, the second coverslip was quickly placed on top of the spacer and gelation was continued for 5 min. Upon completion of the gelation process, both parts of the mold were carefully removed and the cell-seeded hydrogel was transferred to a tissue culture dish (diameter, 35 mm, Greiner). Hydrogels were submerged in 2 ml DMEM and incubated at 37°C in a humidified atmosphere containing 5% CO_2_.

#### Bioluminescence intensity measurements

Cultivation media were replaced with DMEM supplemented with 47 µM luciferin. The validation setup was transferred to the imaging platform of an *in vivo* imaging system (IVIS 100, Perkin-Elmer, USA). Images were taken with a 1-inch CCD camera cooled to −105°C. The field of view (FOV) was set to 10×10 cm. Image sequences (acquisition time, 1 min) were acquired with 5 min intervals during a scanning period of 3 hours, 10 min intervals during 5 hours, and 30 min intervals during 4 hours. Images were processed in the LivingImage software (Perkin-Elmer) and radiance units refer to the number of photons per second that are leaving a square centimeter of the h and radiating into a solid angle of one steradian (Ω, fraction of the isotropic radiation field which can be thought of as a 3D cone of light emitted from the surface). Measurements were performed at 37°C.

#### Single-cell bioluminescence microscopy

After luciferin substrate addition, the validation setup was transferred to the stage of luminescence microscope (LV 200, Olympus). Bioluminescence was imaged with a UPLSAPO 60× oil objective (NA: 1.35) and transmitted to a cooled CCD camera. Time-lapse images were collected with 5 s acquisition times and EM gain was set at 1200× (photon imaging mode, Hamamatsu) Active cells were imaged at the central hydrogel position within a confocal volume at the maximal working distance (i.e. 150 µm) from the glass coverslip.

### Quantification of Active Cell Numbers in Agarose Gels

Viable cell numbers were quantified using the Live/Dead viability/cytotoxicity kit (Invitrogen). Agarose hydrogels were rinsed with PBS solution, covered with Live/Dead staining solution containing 2 µM calcein AM and 4 µM ethidium homodimer-1 in PBS, and incubated for 1 hour in the dark. The dye solution was discarded afterwards and background due to residual stain was washed away with PBS. Live and dead cell numbers were counted via a sphere fitting algorithm in Imaris Bitplane (Zurich, Switzerland). DNA content was evaluated according to a protocol described by Grayson et al. [40] Briefly, agarose hydrogels were washed in PBS, transferred to 400 µl of digestion buffer (10 mM Tris, 1 mM EDTA, and 0.1% Triton X-100) with 0.1 mg/ml proteinase K in centrifugation tubes, and incubated at 56°C for 3 hours. Supernatant was collected after removal of debris by centrifugation (13,000 rpm, 1 min) and measured with a Qubit system (Invitrogen).

### Visualization of Cell Mitochondria

293T cells were seeded on PLL coated glass coverslips (200,000 cells per slide). Cells were incubated overnight prior to staining. Cell mitochondria were stained with a 200 nM MitoTracker dye concentration (MitoTracker Red CM-H2Xros, Invitrogen) in DMEM for 20 min in the dark. The staining solution was supplemented with Na_2_SO_3_ (1%) to expose the cells to hypoxia. Cell nuclei were stained by addition of the cell permeable nucleic acid stain, Hoechst (Invitrogen). After the staining was completed, adherent cells were washed with pre-warmed DMEM. Cells were subsequently fixed by medium replacement with DMEM containing 2% formaldehyde at 37°C (30 min). After fixation, the cells were rinsed with PBS and stored in PBS for visualization. Cell mitochondria were visualized on an inverted confocal microscope (FV1000, Olympus) with a UPLSAPO 100× oil objective (NA: 1.4). Image stacks were acquired with a pixel size of 73×73 nm and consisted of 1024×1024 pixels.

## Supporting Information

Figure S1 **293T cells contain an average firefly luciferase concentration of 0.917 amol cell^−1^ that is constitutively expressed during cultivation.** (**A**) Total emitted photon flux from recombinant luciferase (Rluc, *R^2^* = 0.99) and from firefly luciferase extracted from 293T cells (fLuc, *R^2^* = 1) were used to obtain a standard curve of luciferase concentration versus active cell number. Luciferase activity was measured in Luciferase Assay Reagent (Promega). (**B**) Time-dependent stability of cellular luciferase concentration during cell culture. Error bars, ±1 s.d. unit; n ≥3.
(TIF)

Figure S2 (**A**) Intrinsic diffusion rates of D-luciferin through 2% agarose gels in absence or presence of cells (1×10^6^ cells ml^−1^) obtained from concentration gradient dependent diffusion measurements. (**B**) Fluorescein tracer self-diffusion rates in Dulbecco’s Modified Eagle Medium (DMEM) or in 2% agarose gels measured by Fluorescence Recovery After Photobleaching (FRAP). All measurements were performed at 37°C incubation temperature. Error bars, ±1 s.d. unit; n ≥4. Results were declared significant (indicated by an asterisk) if the p-value was less than 0.05.
(TIF)

Figure S3 (**A**) Diffusion rates of Rhodamine B fluorescent tracer molecules in 2% agarose gels at various tracer concentrations with an average diffusion rate of 338 µm^2^ s^−1^ (dotted line) determined by Fluorescence Correlation Spectroscopy (FCS). Reference values for Rhodamine 6G tracer diffusion in DMEM are also shown. The experimentally obtained autocorrelation curves were best fitted with a function that accounts for anomalous subdiffusion, presumably induced by macromolecular crowding [32,48]. (**B**) Anomality parameter for tracer diffusion in agarose or in DMEM. As expected from the small size of fluorescent tracer molecules and the low agarose concentration, we did not observe significant changes in anomality. All measurements were performed at RT (21°C). Error bars, ±1 s.d. unit; n = 5.
(TIF)

Figure S4 **Fluorescent oxygen sensitive microbeads (OSB) were integrated in agarose gels to measure radial gradients in oxygen concentration.** Scanning electron microscopy (SEM) of OSB before (**A,B**) and after (**C,D**) coating with a silica shell. Cross-sections of the silica beads were obtained by Focused Ion Beam (FIB) ablation. (**C**) Imaging of OSB after mechanical grinding reveals presence of silica shell. Scale bar, 5 µm. (**E**) Calibration of OSB in DMEM at saturated or hypoxic oxygen conditions. (F) Radial oxygen profiles from oxygen saturated agarose gels measured with OSB (5×10^6^ beads ml^−1^) obtained after different incubation periods. Error bars, ±1 s.d. unit; n = 3. (**G**) Evolution of time-dependent oxygen consumption rates (OCR) of 293T cells embedded in 2% agarose gels. Cell OCR are obtained from profile fitting of the oxygen model simulations to the radial oxygen profiles measured with OSB.
(TIF)

Figure S5 **Measurement of active cell numbers present in agarose hydrogels.** (**A**) Cell densities obtained from DNA measurements on extracted cell lysate solutions. (**B**) Spatial distribution of cell viability quantified by the Live/Dead viability assay. Radial positions are representative for the regions identified in (**C**). Gels are imaged with an inverted microscope from bottom position, along the radial direction at day 1 (**C**), day 2 (**D**), and day 3 (**E**). For all cultivation times, a peripheral region is visible with decreased cell viability (region 4). Images show a z-stack projection of live (green) and dead (red) cells. Error bars, ±1 s.d. unit; n ≥4. Scale bar, 1 mm.
(TIF)

Figure S6 **Quantitative analysis of bioluminescence signal peak behavior and correlation with available oxygen concentration.** Analyses were performed on signals obtained from entire gels (A–D) or from local measurements in the hydrogel center (E–H). Initial peak behavior was described by the peak position (A,E) and FHWM (B,F). (C,G) Decay rates of bioluminescent light emission were obtained from exponential curve fitting. (D,H) Time-dependent evolution of oxygen concentration described by the mathematical bioluminescence-oxygen model (solid line) with minimum and maximum values (dotted lines), and average oxygen concentrations measured with OSB (grey dots). Error bars, ±1 s.d. unit; n ≥3.
(TIF)

Figure S7 **2D profiles of luciferin spatial distribution in cell-seeded agarose gels.** Distribution profiles are obtained from the bioluminescence-oxygen model and indicate luciferin concentration gradients at the bioluminescence peak positions. Profiles at the left side from the dashed lines represent intracellular luciferin concentrations at the experimentally defined peak position and profiles at the right side show intracellular luciferin concentrations at the model peak positions. (Initial extracellular luciferin concentration, 47 µM).
(TIF)

Figure S8 (**A**) Fluorescence imaging of a luciferin concentration gradient established between a saturated solution (left) and a tracer-free 293T cell-seeded agarose gel (right). Positions occupied by the cells appear as dark spots (red arrowheads). (**B**) Multichannel fluorescence image shows cells embedded in the agarose (green) in combination with diffusing luciferin (red) and reacted oxyluciferin (blue). Scale bar, 400 µm.
(TIF)

Table S1 **Overview of the parameter values implemented in the bioluminescence-oxygen model.**
(DOCX)

Text S1 **Implementation of the mathematical bioluminescence-oxygen model.**
(DOCX)

Video S1 **2D bioluminescence profiles of cell-seeded agarose gels imaged from top position (hydrogel diameter, 8 mm).** Profiles are imaged with an IVIS 100 imaging system. Radiance units are in p s^−1^ cm^−2^.
(AVI)

## References

[1] Gilbert PM, Havenstrite KL, Magnusson KE, Sacco A, Leonardi NA, et al (2010) Substrate elasticity regulates skeletal muscle stem cell self-renewal in culture. Science 329: 1078–81. 20647425 10.1126/science.1191035 PMC2929271

[2] Weiss MS, Bernabe BP, Bellis AD, Broadbelt LJ, Jeruss JS, et al (2010) Dynamic, Large-Scale Profiling of Transcription Factor Activity from Live Cells in 3D Culture. PLoS One 5: e14026. 21103341 10.1371/journal.pone.0014026 PMC2984444

[3] Dunn CA, Jin QM, Taba M, Franceschi RT, Rutherford RB, et al (2005) BMP gene delivery for alveolar bone engineering at dental implant defects. Mol Ther 11: 294–9. 15668141 10.1016/j.ymthe.2004.10.005 PMC2573463

[4] McMillin DW, Delmore J, Weisberg E, Negri JM, Geer DC, et al (2010) Tumor cell-specific bioluminescence platform to identify stroma-induced changes to anticancer drug activity. Nat Med 16: 483–U171. 20228816 10.1038/nm.2112 PMC3786785

[5] Roda A, Guardigli M, Michelini E, Mirasoli M (2009) Bioluminescence in analytical chemistry and in vivo imaging. Trends Anal Chem 28: 307–22.

[6] Badr CE, Tannous BA (2011) Bioluminescence imaging: progress and applications. Trends Biotechnol 29: 624–33. 21788092 10.1016/j.tibtech.2011.06.010 PMC4314955

[7] Ankrum J, Karp JM (2010) Mesenchymal stem cell therapy: Two steps forward, one step back. Trends Mol Med 16: 203–9. 20335067 10.1016/j.molmed.2010.02.005 PMC2881950

[8] Keyaerts M, Caveliers V, Lahoutte T (2010) Bioluminescence imaging: looking beyond the light. Trends Mol Med 18: 164–72. 10.1016/j.molmed.2012.01.005 22321645

[9] Khalil AA, Jameson MJ, Broaddus WC, Lin PS, Dever SM, et al (2013) The Influence of Hypoxia and pH on Bioluminescence Imaging of Luciferase-Transfected Tumor Cells and Xenografts. International journal of molecular imaging 2013: 287697. 23936647 10.1155/2013/287697 PMC3723249

[10] Moriyama EH, Niedre MJ, Jarvi MT, Mocanu JD, Moriyama Y, et al (2008) The influence of hypoxia on bioluminescence in luciferase-transfected gliosarcoma tumor cells in vitro. Photochem Photobiol Sci 7: 675–80. 18528551 10.1039/b719231b

[11] Timmins GS, Robb FJ, Wilmot CM, Jackson SK, Swartz HM (2001) Firefly flashing is controlled by gating oxygen to light-emitting cells. J Exp Biol 204: 2795–801. 11683435 10.1242/jeb.204.16.2795

[12] Hagen T, Taylor CT, Lam F, Moncada S (2003) Redistribution of intracellular oxygen in hypoxia by nitric oxide: Effect on HIF1 alpha. Science 302: 1975–8. 14671307 10.1126/science.1088805

[13] Ivanovic Z (2009) Hypoxia or in situ normoxia: The stem cell paradigm. J Cell Physiol 219: 271–5. 19160417 10.1002/jcp.21690

[14] Gandelman O, Allue I, Bowers K, Cobbold P (1994) Cytoplasmic Factors That Affect the Intensity and Stability of Bioluminescence from Firefly Luciferase in Living Mammalian-Cells. J Biolum Chemilum 9: 363–71. 10.1002/bio.1170090603 7879652

[15] Fraga H (2008) Firefly luminescence: a historical perspective and recent developments. Photochem Photobiol Sci 7: 146–58. 18264582 10.1039/b719181b

[16] Zhang Y, Bressler JP, Neal J, Lal B, Bhang HE, et al (2007) ABCG2/BCRP expression modulates D-Luciferin based bioluminescence imaging. Cancer Res 67: 9389–97. 17909048 10.1158/0008-5472.CAN-07-0944

[17] Fukuda R, Zhang H, Kim JW, Shimoda L, Dang CV, et al (2007) HIF-1 regulates cytochrome oxidase subunits to optimize efficiency of respiration in hypoxic cells. Cell 129: 111–22. 17418790 10.1016/j.cell.2007.01.047

[18] Berthold F, Hennecke M, Wulf J (2011) Instrumentation for Chemiluminescence and Bioluminescence. In: Roda A, editor. Chemiluminescence and Bioluminescence: Past, Present and Future. Cambridge, UK: Royal Society of Chemistry. 113–39.

[19] Denburg JL, Lee RT, Mcelroy WD (1969) Substrate-Binding Properties of Firefly Luciferase.I. Luciferin-Binding Site. Arch Biochem Biophys 134: 381–394. 5354768 10.1016/0003-9861(69)90297-5

[20] Ignowski JM, Schaffer DV (2004) Kinetic analysis and modeling of firefly luciferase as a quantitative reporter gene in live mammalian cells. Biotechnol Bioeng 86: 827–34. 15162459 10.1002/bit.20059

[21] Schnell S, Turner TE (2004) Reaction kinetics in intracellular environments with macromolecular crowding: simulations and rate laws. Prog Biophys Mol Biol 85: 235–60. 15142746 10.1016/j.pbiomolbio.2004.01.012

[22] Minton AP (2006) How can biochemical reactions within cells differ from those in test tubes? J Cell Sci 119: 2863–9. 16825427 10.1242/jcs.03063

[23] Shinde R, Perkins J, Contag CH (2006) Luciferin derivatives for enhanced in vitro and in vivo bioluminescence assays. Biochemistry-Us 45: 11103–12. 10.1021/bi060475o 16964971

[24] Fraga H, Fernandes D, Fontes R, da Silva JCGE (2005) Coenzyme A affects firefly luciferase luminescence because it acts as a substrate and not as an allosteric effector. Febs J 272: 5206–16. 16218952 10.1111/j.1742-4658.2005.04895.x

[25] Jonsson P, Jonsson MP, Tegenfeldt JO, Hook F (2008) A method improving the accuracy of fluorescence recovery after photobleaching analysis. Biophys J 95: 5334–48. 18567628 10.1529/biophysj.108.134874 PMC2586554

[26] Leskovac V (2003) Chemical Kinetics. Comprehensive Enzyme Kinetics. New York: Kluwer Academic/Plenum Publishers. 11–30.

[27] Kopelman R (1988) Fractal reaction kinetics. Science 241: 1620–6. 17820893 10.1126/science.241.4873.1620

[28] Lambrechts D, Roeffaers M, Kerckhofs G, Roberts SJ, Hofkens J, et al (2013) Fluorescent oxygen sensitive microbead incorporation for measuring oxygen tension in cell aggregates. Biomaterials 34: 922–9. 23122803 10.1016/j.biomaterials.2012.10.019

[29] Mohyeldin A, Garzon-Muvdi T, Quinones-Hinojosa A (2010) Oxygen in Stem Cell Biology: A Critical Component of the Stem Cell Niche. Cell Stem Cell 7: 150–61. 20682444 10.1016/j.stem.2010.07.007

[30] Peerani R, Zandstra PW (2010) Enabling stem cell therapies through synthetic stem cell-niche engineering. J Clin Invest 120: 60–70. 20051637 10.1172/JCI41158 PMC2798701

[31] Müller C, Loman A, Pacheco V, Koberling F, Willbold D, et al (2008) Precise measurement of diffusion by multi-color dual-focus fluorescence correlation spectroscopy. EPL (Europhysics Letters) 83: 46001.

[32] Weiss M, Elsner M, Kartberg F, Nilsson T (2004) Anomalous subdiffusion is a measure for cytoplasmic crowding in living cells. Biophys J 87: 3518–24. 15339818 10.1529/biophysj.104.044263 PMC1304817

[33] Shimomura O (2006) Bioluminescence: Chemical Principles and Methods. 5 Toh Tuck Link, Singapore 596224: World Scientific Publishin Co Pte Ltd. 470 p.

[34] Gandelman O, Brovko LY, Ugarova N, Chikishev AY, Shkurimov A (1993) Oxyluciferin fluorescence is a model of native bioluminescence in the firefly luciferin–luciferase system. Journal of Photochemistry and Photobiology B: Biology 19: 187–91.

[35] Crank J (1979) Infinite and Semi-infinite Media. The mathematics of diffusion: Oxford university press. 28–43.

[36] Graf C, Vossen DL, Imhof A, van Blaaderen A (2003) A general method to coat colloidal particles with silica. Langmuir 19: 6693–700. 10.1021/la100188w 20334440

[37] Chen S-L, Dong P, Yang G-H, Yang J-J (1996) Characteristic aspects of formation of new particles during the growth of monosize silica seeds. J Colloid Interface Sci 180: 237–41.

[38] Bogush G, Tracy M, Zukoski C (1988) Preparation of monodisperse silica particles: control of size and mass fraction. J Non-Cryst Solids 104: 95–106.

[39] Masalov VM, Sukhinina NS, Kudrenko EA, Emelchenko GA (2011) Mechanism of formation and nanostructure of Stober silica particles. Nanotechnology 22: 275718. 21613739 10.1088/0957-4484/22/27/275718

[40] Grayson WL, Frohlich M, Yeager K, Bhumiratana S, Chan ME, et al (2010) Engineering anatomically shaped human bone grafts. Proc Natl Acad Sci USA 107: 3299–304. 19820164 10.1073/pnas.0905439106 PMC2840502

