# Correction: A Causal Relation between Bioluminescence and Oxygen to Quantify the Cell Niche

**DOI:** 10.1371/journal.pone.0128852

**Published:** 2015-05-28

**Authors:** Dennis Lambrechts, Maarten Roeffaers, Karel Goossens, Johan Hofkens, Greetje Vande Velde, Tom Van de Putte, Jan Schrooten, Hans Van Oosterwyck

Dr. Greetje Vande Velde should be included in the author byline instead of the Acknowledgments. She should be listed as the fifth author, and her affiliations are: Biomedical MRI Unit, Department of Imaging and Pathology, Faculty of Medicine, KU Leuven, Leuven, Belgium and the Molecular Small Animal Imaging Centre, Department of Imaging and Pathology, Faculty of Medicine, KU Leuven, Leuven, Belgium.

The correct citation is: Lambrechts D, Roeffaers M, Goossens K, Hofkens J, Vande Velde G, Van de Putte T, et al. (2014) A Causal Relation between Bioluminescence and Oxygen to Quantify the Cell Niche. PLoS ONE 9(5): e97572. doi:10.1371/journal.pone.0097572

The Acknowledgments should read: We thank Tom Van der Donck for his expert assistance with the SEM/FIB and prof. Geert Carmeliet for providing the MT staining. Viral vectors were constructed and produced in the Leuven Viral Vector Core (LVVC). We acknowledge the Olympus Corporation for providing the LV200 on loan. This work is part of Prometheus, the Leuven Research & Development Division of Skeletal Tissue Engineering of the KU Leuven (www.kuleuven.be/prometheus).

There is an error in the first sentence of the second paragraph of the “293T Cell Culture and Transduction” subsection of the Materials and Methods. The correct sentence is: 293T cells were transduced with a lentiviral vector (pCH-EF1a-3flag-fLuc-T2A-eGFP-Ires-Bsd, 3.16×10^8^ TU ml^-1^).
