# Supplementary figures and images for: A Causal Relation between Bioluminescence and Oxygen to Quantify the Cell Niche
Source: PLoS One. 2014 May 19;9(5):e97572. doi: 10.1371/journal.pone.0097572 (PMC4026314; doi:10.1371/journal.pone.0097572)

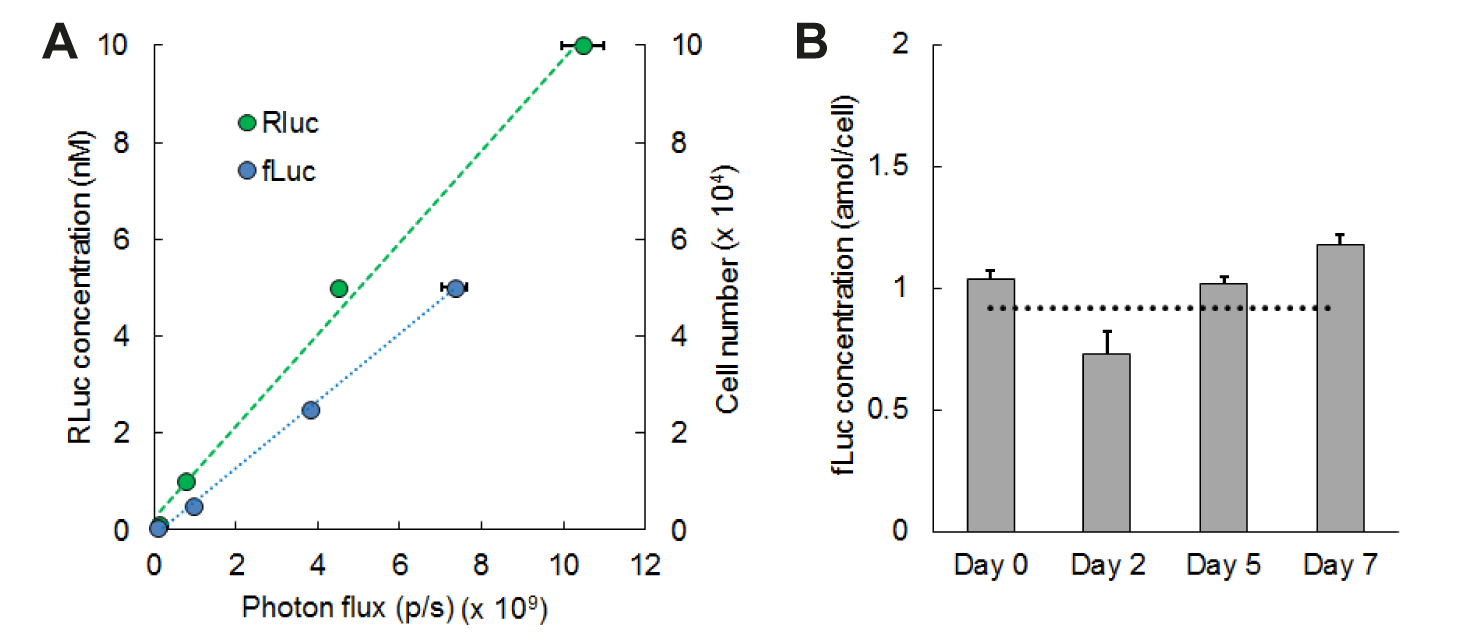

Supplement: Figure S1 — 293T cells contain an average firefly luciferase concentration of 0.917 amol cell−1 that is constitutively expressed during cultivation. (A) Total emitted photon flux from recombinant luciferase (Rluc, R2 = 0.99) and from firefly luciferase extracted from 293T cells (fLuc, R2 = 1) were used to obtain a standard curve of luciferase concentration versus active cell number. Luciferase activity was measured in Luciferase Assay Reagent (Promega). (B) Time-dependent stability of cellular luciferase concentration during cell culture. Error bars, ±1 s.d. unit; n ≥3. (TIF) [file pone.0097572.s001.tif]

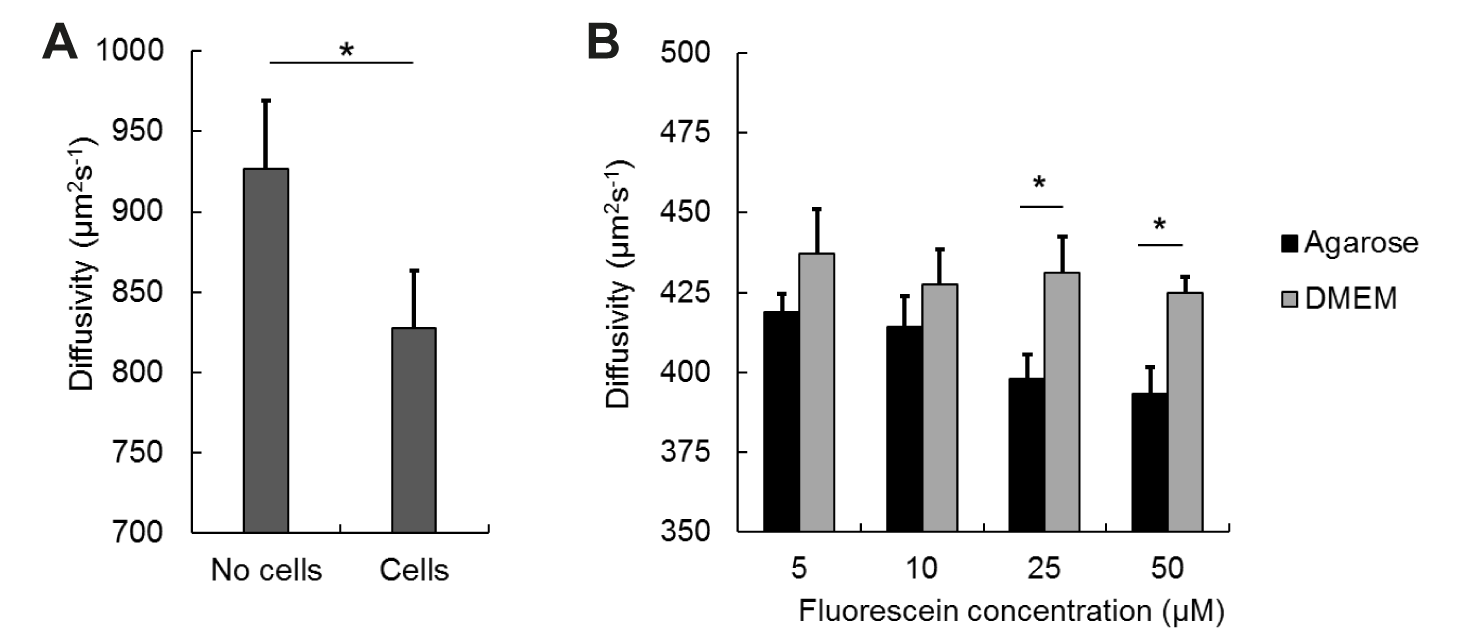

Supplement: Figure S2 — (A) Intrinsic diffusion rates of D-luciferin through 2% agarose gels in absence or presence of cells (1×10^6^ cells ml−1) obtained from concentration gradient dependent diffusion measurements. (B) Fluorescein tracer self-diffusion rates in Dulbecco’s Modified Eagle Medium (DMEM) or in 2% agarose gels measured by Fluorescence Recovery After Photobleaching (FRAP). All measurements were performed at 37°C incubation temperature. Error bars, ±1 s.d. unit; n ≥4. Results were declared significant (indicated by an asterisk) if the p-value was less than 0.05. (TIF) [file pone.0097572.s002.tif]

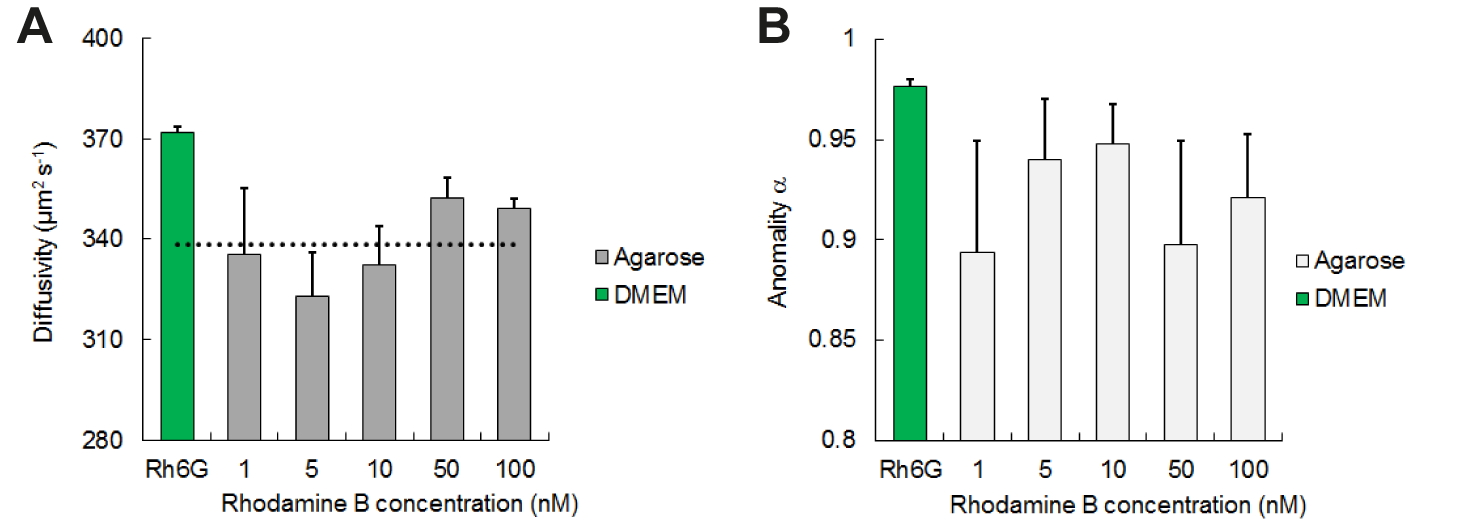

Supplement: Figure S3 — (A) Diffusion rates of Rhodamine B fluorescent tracer molecules in 2% agarose gels at various tracer concentrations with an average diffusion rate of 338 µm2 s−1 (dotted line) determined by Fluorescence Correlation Spectroscopy (FCS). Reference values for Rhodamine 6G tracer diffusion in DMEM are also shown. The experimentally obtained autocorrelation curves were best fitted with a function that accounts for anomalous subdiffusion, presumably induced by macromolecular crowding [32,48]. (B) Anomality parameter for tracer diffusion in agarose or in DMEM. As expected from the small size of fluorescent tracer molecules and the low agarose concentration, we did not observe significant changes in anomality. All measurements were performed at RT (21°C). Error bars, ±1 s.d. unit; n = 5. (TIF) [file pone.0097572.s003.tif]

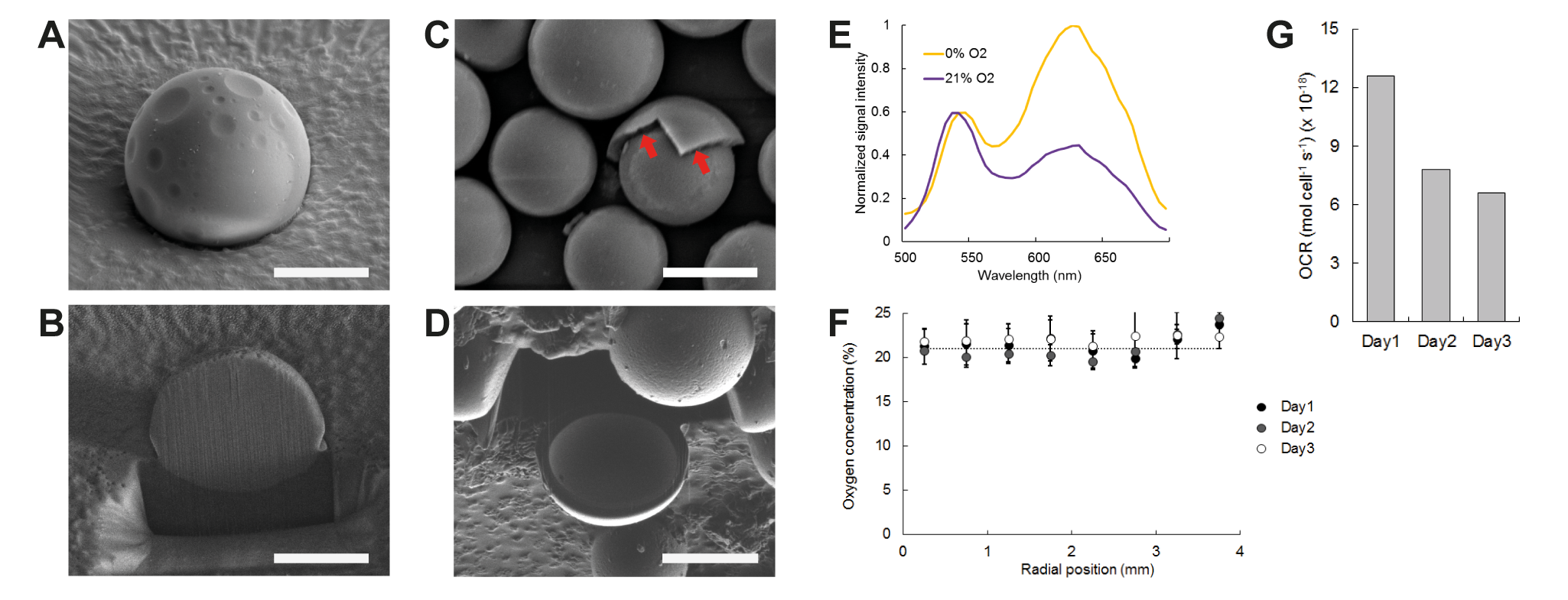

Supplement: Figure S4 — Fluorescent oxygen sensitive microbeads (OSB) were integrated in agarose gels to measure radial gradients in oxygen concentration. Scanning electron microscopy (SEM) of OSB before (A,B) and after (C,D) coating with a silica shell. Cross-sections of the silica beads were obtained by Focused Ion Beam (FIB) ablation. (C) Imaging of OSB after mechanical grinding reveals presence of silica shell. Scale bar, 5 µm. (E) Calibration of OSB in DMEM at saturated or hypoxic oxygen conditions. (F) Radial oxygen profiles from oxygen saturated agarose gels measured with OSB (5×10^6^ beads ml−1) obtained after different incubation periods. Error bars, ±1 s.d. unit; n = 3. (G) Evolution of time-dependent oxygen consumption rates (OCR) of 293T cells embedded in 2% agarose gels. Cell OCR are obtained from profile fitting of the oxygen model simulations to the radial oxygen profiles measured with OSB. (TIF) [file pone.0097572.s004.tif]

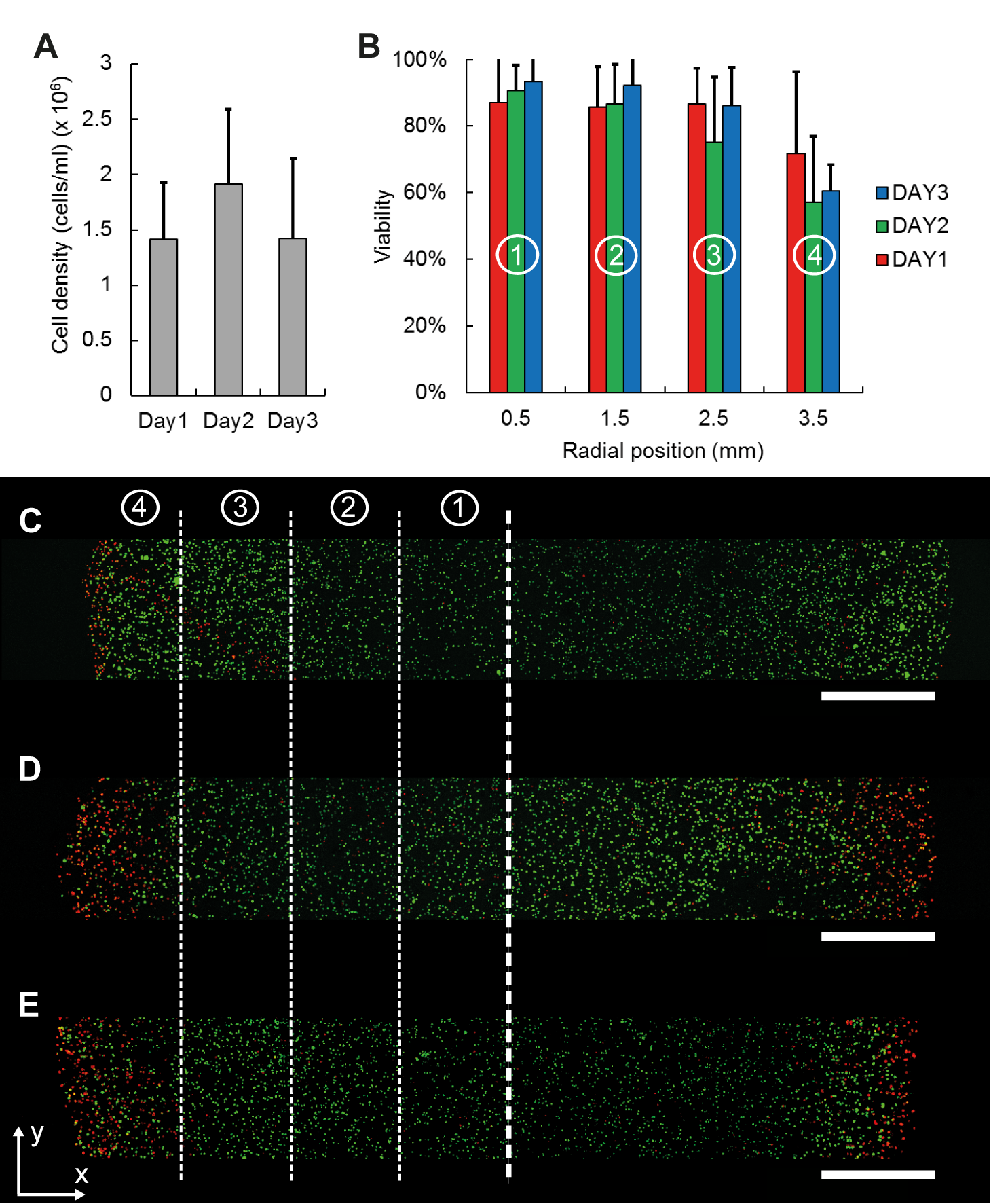

Supplement: Figure S5 — Measurement of active cell numbers present in agarose hydrogels. (A) Cell densities obtained from DNA measurements on extracted cell lysate solutions. (B) Spatial distribution of cell viability quantified by the Live/Dead viability assay. Radial positions are representative for the regions identified in (C). Gels are imaged with an inverted microscope from bottom position, along the radial direction at day 1 (C), day 2 (D), and day 3 (E). For all cultivation times, a peripheral region is visible with decreased cell viability (region 4). Images show a z-stack projection of live (green) and dead (red) cells. Error bars, ±1 s.d. unit; n ≥4. Scale bar, 1 mm. (TIF) [file pone.0097572.s005.tif]

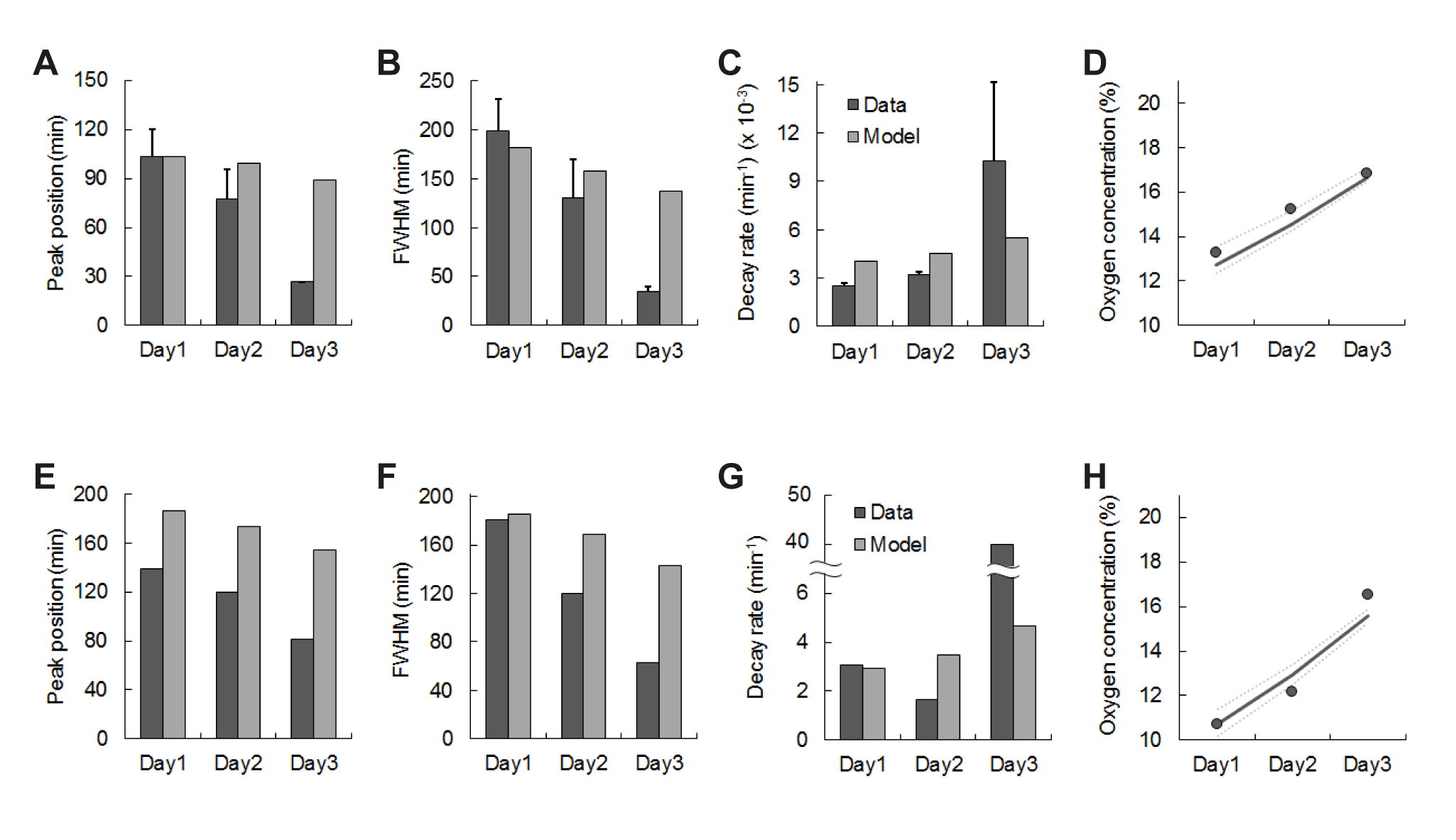

Supplement: Figure S6 — Quantitative analysis of bioluminescence signal peak behavior and correlation with available oxygen concentration. Analyses were performed on signals obtained from entire gels (A–D) or from local measurements in the hydrogel center (E–H). Initial peak behavior was described by the peak position (A,E) and FHWM (B,F). (C,G) Decay rates of bioluminescent light emission were obtained from exponential curve fitting. (D,H) Time-dependent evolution of oxygen concentration described by the mathematical bioluminescence-oxygen model (solid line) with minimum and maximum values (dotted lines), and average oxygen concentrations measured with OSB (grey dots). Error bars, ±1 s.d. unit; n ≥3. (TIF) [file pone.0097572.s006.tif]

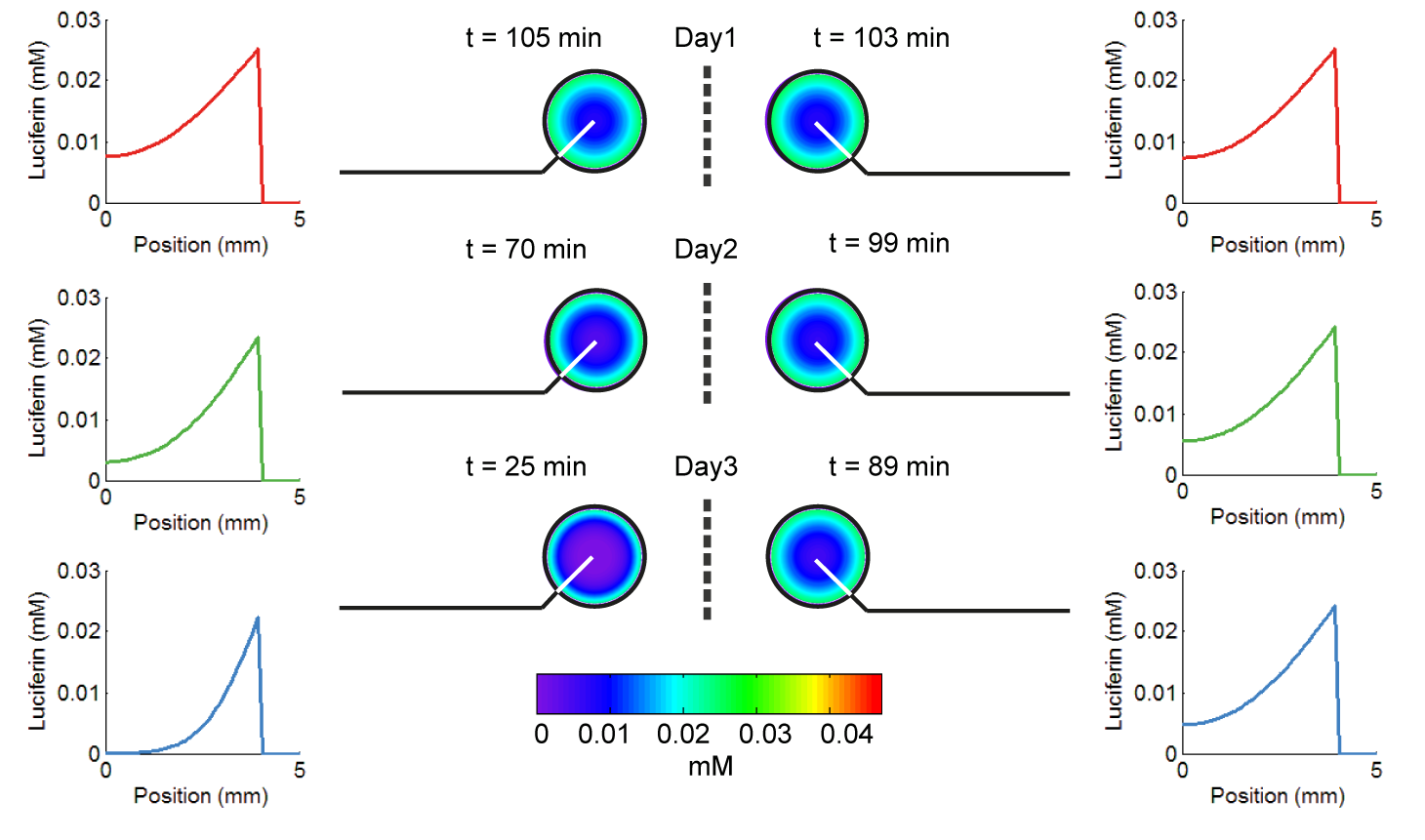

Supplement: Figure S7 — 2D profiles of luciferin spatial distribution in cell-seeded agarose gels. Distribution profiles are obtained from the bioluminescence-oxygen model and indicate luciferin concentration gradients at the bioluminescence peak positions. Profiles at the left side from the dashed lines represent intracellular luciferin concentrations at the experimentally defined peak position and profiles at the right side show intracellular luciferin concentrations at the model peak positions. (Initial extracellular luciferin concentration, 47 µM). (TIF) [file pone.0097572.s007.tif]

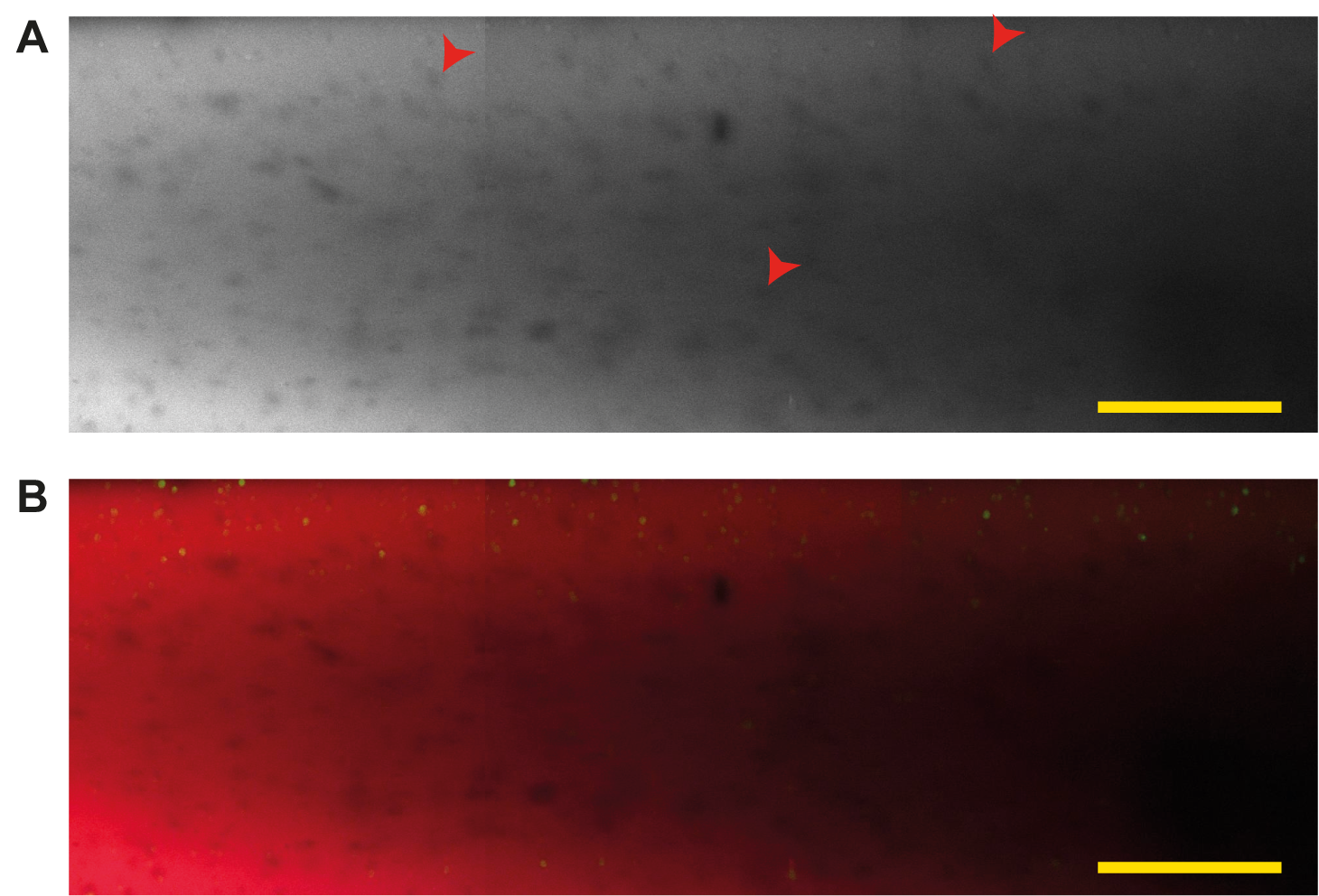

Supplement: Figure S8 — (A) Fluorescence imaging of a luciferin concentration gradient established between a saturated solution (left) and a tracer-free 293T cell-seeded agarose gel (right). Positions occupied by the cells appear as dark spots (red arrowheads). (B) Multichannel fluorescence image shows cells embedded in the agarose (green) in combination with diffusing luciferin (red) and reacted oxyluciferin (blue). Scale bar, 400 µm. (TIF) [file pone.0097572.s008.tif]
