# Supplementary material for: A Causal Relation between Bioluminescence and Oxygen to Quantify the Cell Niche
Source: PLoS One. 2014 May 19;9(5):e97572. doi: 10.1371/journal.pone.0097572 (PMC4026314; doi:10.1371/journal.pone.0097572)
Supplement: Table S1 — Overview of the parameter values implemented in the bioluminescence-oxygen model. (DOCX) [file pone.0097572.s009.docx]

Table S1. Overview of the parameter values implemented in the bioluminescence-oxygen model.

| **Model Parameter** | **Value** | **Unit** | **Reference** | |
| --- | --- | --- | --- | --- |
| *Luciferin oxidation (living cells)* | | | | |
| *k_cat_* (catalytic rate constant, CRC) | 1.1 x 10^-3^ | mol olc / (mol luc · s) ^†^ | | Fitted  (Fig. 2E) |
| *k_cat,21,1_* (CRC, 21% O_2_) | 1 | - | |  |
| *k_cat21,2_* (CRC, 21% O_2_) | 1.01 x 10^-1^ | - | |  |
| *k_cat,0_* (CRC, 0% O_2_) | 6 x 10^-3^ | - | |  |
| *I_21,1_* (exponential decay constant, 21% O_2_) | -180 | s^-1^ | | Measured  (Fig. 2E) |
| *I_21,2_* | -27 | s^-1^ | | Measured  (Fig. 2E) |
| *I_0_* (exponential decay constant, 0% O_2_) | -3 | s^-1^ | | Measured  (Fig. 2E) |
| *k_d,Dluc_* (luciferin decay constant) | 4.83 x 10^-6^ | s^-1^ | | Ignowski et al. [20] |
| *Γ* (Molecular crowding factor) | 1.5 | - | | Fitted  (Fig. 2E) |
| *κ_21_* (transition threshold, 21% O_2_) | 0.1 | - | | Fitted  (Fig. 2E) |
| *κ_0_* (transition threshold, 0% O_2_) | 15 | - | | Fitted  (Fig. 2E) |
| *Luciferin oxidation (recombinant luciferase)* | | | | |
| \| *k_cat_* (catalytic rate constant, CRC) \| 1.12 x 10^-1^ \| mol olc /  (mol luc · s) \| Fitted  (Fig. 2A,B) \| \| --- \| --- \| --- \| --- \| \| *k_cat,21_* (CRC, 21% O_2_) \| 1 \| - \|  \| \| *k_cat0_* (CRC, 0% O_2_) \| 0.18 \| - \|  \| \| *S_21_* \| 60 \| - \| Measured  (Fig. 2A,B) \| \| *I_21_* (exponential decay constant,  21% O_2_) \| -3948 \| s^-1^ \| Measured  (Fig. 2A,B) \| \| *S_0_* (exponential decay constant,  0% O_2_) \| 382 \| - \| Measured  (Fig. 2A,B) \| \| *I_0_* \| -1452 \| - \|  \| \| *K_m,21_* (Michaelis constant, 21% O_2_) \| 4.26 x 10^-2^ \| mol m^-3^ \| Measured (Fig. 2A,B) \| \| *K_m,0_* (Michaelis constant, 0% O_2_) \| 9.38 x 10^-1^ \| mol m^-3^ \| Measured (Fig. 2A,B) \| | | | | |
| *Luciferin diffusion* | | | | |
| *D_Dluc,A_* (diffusivity in agarose) | 9.27 x 10^-10^ | m^2^ s^-1^ | | Measured  (Fig. S2) |
| *D_Dluc,w_* (diffusivity in DMEM) | 1.00 x 10^-9^ | m^2^ s^-1^ | | Measured  (Fig. S2) |
| *D_Dluc,m_* (diffusivity in cell memebrane) | 8 x 10^-11^ | m^2^ s^-1^ | | Fitted  (Fig. 2E) |
| *λ_c_* (thickness cell membrane) | 4 x 10^-9^ | m | | Ignowski et al. [20] |
| *A_c_* (Surface area cell membrane) | 3.14 x 10^-10^ | m^2^ | | 10 μm sphere |
| *Luciferase production* | | | | |
| *k_s,luc_* (luciferase synthesis rate) | 7.64 x 10^-23^ | mol cell^-1^s^-1^ | | Calculated for constitutive promotor (i.e. equals *k_d,luc_ · c_luc_*) |
| *k_d,luc_* (luciferase decay constant) | 8.33 x 10^-5^ | s^-1^ | | Ignowski et al. [20] |
| *Light production* | | | | |
| *RLU* (recombinant luciferase) | 1.38 x 10^7^ | p s^-1^ cm^-2^ / (mol olc) | | Measured  (Fig. 2A,B) |
| *RLU (*living cells) | 1.38 x 10^7^ | p s^-1^ cm^-2^ / (mol olc) | |  |
| *Oxygen diffusion* | | | | |
| *D_O2,w_* (diffusivity in water) | 3 x 10^-9^ | m^2^ s^-1^ | | Demol et al. [49] |
| *D_O2,a_* (diffusivity in agarose) | 1 x 10^-9^ | m^2^ s^-1^ | | Hulst et al. [45] |
|  |  |  |  | |

Note: ^†^olc, oxyluciferin complex.
