# Supplementary material for: A Causal Relation between Bioluminescence and Oxygen to Quantify the Cell Niche
Source: PLoS One. 2014 May 19;9(5):e97572. doi: 10.1371/journal.pone.0097572 (PMC4026314; doi:10.1371/journal.pone.0097572)
Supplement: Text S1 — Implementation of the mathematical bioluminescence-oxygen model. (DOCX) [file pone.0097572.s010.docx]

Supporting Text S1

Mathematical bioluminescence-oxygen model

Model geometry

We implemented the geometry of the validation setup as one half of a vertical cross-section, taking advantage of the axisymmetry of this setup (Fig. 3B). Three regions were identified in this geometry: hydrogel, radial periphery, and medium. A peripheral region of 500 µm thickness was implemented to account for decreased cell viabilities near the construct surface (Fig. S5). The total modeled geometry had a height of 3.15 mm and radius of 17.5 mm. Further details on the geometrical dimensions can be found in the **Materials and** **Methods** section.

Model equations

Spatiotemporal changes in oxygen availability ($c_{O_{2}}$), luciferin concentration (*c_Dluc_*), intracellular luciferase concentration (*c_luc_*) and bioluminescent light generation (*c_L_*) were mathematically described by a set of five partial differential equations. Variations in intra- and extracellular luciferin concentrations were decoupled and implemented into the model as two separate equations. Extracellular luciferin concentrations changed as a result of concentration gradient induced movement (described by the luciferin diffusivity, *D_Dluc,E_*), active uptake by the cells (membrane diffusion rate, *D_Dluc,m_*), and natural decay of the substrate (lucferin decay rate, *k_Dluc_*).

$\begin{matrix} \frac{\partial c_{Dluc,E}\left(z,r,t\right)}{\partial t}=D_{Dluc,E}\left(z,r,t\right)\cdot\nabla^{2}c_{Dluc,E}\left(z,r,t\right)-\frac{D_{Dluc_{m}} A_{\mathrm{c} }}{\lambda_{c}}\cdot\\ \left(c_{Dluc,E}\left(z,r,t\right)- c_{Dluc,I}\left(z,r,t\right)\right)\cdot c_{cell}\left(z,r,t\right)- k_{Dluc}\cdot c_{Dluc,E}\left(z,r,t\right) \end{matrix}$ (**1**)

Luciferin substrate molecules taken up by the cells are enzymatically converted into oxyluciferin, in a bioluminescence reaction process that is accompanied by the emission of a photon. Luciferin conversion rates are dependent on the available luciferin substrate concentration and can be described by the Michaelis-Menten kinetics. Time-dependent decay of bioluminescent light emission was described by a first order kinetic equation [26]. The straightforward implementation of this equation with a single decay parameter has a clear advantage in describing reactions that undergo exponential decay [41]. Inclusion of this first order kinetic equation with the Michaelis-Menten kinetics also offers a simplified framework for future implementation of inhibitor/activator activity on the bioluminescence reaction process.

$\begin{matrix} \frac{\partial c_{Dluc,I}\left(z,r,t\right)}{\partial t}=\frac{D_{Dluc_{m}} A_{\mathrm{c} }}{\lambda_{c}}\cdot\left(c_{Dluc,E}\left(z,r,t\right)-c_{Dluc,I}\left(z,r,t\right)\right)\cdot c_{cell}\left(z,r,t\right)- \\ k_{Dluc}\cdot c_{Dluc,I}\left(z,r,t\right)-k_{cat}\left(c_{O_{2}}\right)\cdot e^{\sigma\left(t-t_{i}\left(z,r,t\right)\right)}\cdot\frac{c_{Dluc,I}\left(z,r,t\right)}{K_{m}\left(c_{O_{2}}\right)\cdot\Gamma+c_{Dluc,I}\left(z,r,t\right)}\cdot c_{luc} \end{matrix}$ (**2**)

$\frac{\partial c_{L}\left(z,r,t\right)}{\partial t}=k_{cat}\left(c_{O_{2}}\right)\cdot e^{\sigma\left(t-t_{i}\left(z,r,t\right)\right)}\cdot\frac{c_{Dluc,I}\left(z,r,t\right)}{K_{m}\left(c_{O_{2}}\right)\cdot\Gamma+c_{Dluc,I}\left(z,r,t\right)}\cdot c_{luc}\cdot RLU$ (**3**)

Gradients in available oxygen concentration originated from a balance in oxygen uptake rates from the cells (OCR, $Q_{O_{2}}$) and oxygen diffusion rates through the gel (oxygen diffusivity, $D_{O_{2}}$). OCR were also described by a Michaelis-Menten kinetic equation.

$\frac{\partial c_{O_{2}}\left(z,r,t\right)}{\partial t}=D_{O_{2}}\left(z,r,t\right)\cdot\nabla^{2}c_{O_{2}}\left(z,r,t\right)-Q_{O_{2}}\left(z,r,t\right)\cdot c_{cell}\left(z,r,t\right)$ (**4**)

Intracellular firefly luciferase concentrations remained constant during cell cultivation as their expression was under control of a constitutive promoter (Fig. S1B). Changes in average luciferase concentration contained within the construct resulted from corresponding changes in cell density (*c_cell_*).

$\frac{\partial c_{luc}\left(z,r,t\right)}{\partial t}=k_{s,luc}\cdot c_{cell}\left(z,r,t\right)- k_{d,luc}\cdot c_{luc}\left(z,r,t\right)$ (**5**)

These coupled partial differential equations were solved numerically using a previously developed finite volume code. The grid size was 100 µm in axial and radial direction for all three regions. The axisymmetric geometry was directly taken into account by the numerical algorithm and we used the ROWMAP code for time integration [42].

Extracellular luciferin diffusion

Luciferin substrate was injected into the medium surrounding the hydrogel and by the action of concentration gradient induced motility these molecules were able to interact with the agarose-embedded cells. Diffusion rates through agarose hydrogels were obtained from dedicated time-lapse diffusion experiments in absence or presence of cells. From these experiments we observed that seeded cells significantly hindered luciferin transport (Fig. S2A). Enzymatic luciferin conversion in the cells (Table S1) did not have a significant effect on the observed drop in diffusivity which we quantified by implementing a consumption term in the time-lapse diffusion model in COMSOL. Also the obstruction of luciferin movement caused by the seeded cells could not explain these discrepancies, as cells only occupied a volume fraction of ~5 x 10^-4^ in the agarose gel [43]. Therefore, we reasoned that luciferin binding as substrate for the ABC transporter activity could explain these differences in luciferin diffusivity [16]. The average luciferin diffusion rate in DMEM was estimated from relative differences in self-diffusivity between agarose and DMEM measured by FRAP analysis (Fig. S2B).

Intracellular luciferin uptake

A simplified, two-state kinetic model has been previously reported to describe mechanistic features of the ABC transporter activity for luciferin uptake [44]. However, detailed information on rate and dissociation constants is required for practical application of this model, which is currently not available in literature. Therefore we implemented a diffusion model that was shown to be successful in describing long-term (hours) luciferase activity in living cells [20]. Estimations for the cellular luciferin influx were obtained from fitting a membrane diffusion model through the short term dynamic time point measurements of luciferase activity in intact cells (Fig. 2D). This resulted in a value of 8 x 10^-11^ m^2^ s^-1^ for luciferin transmembrane diffusivity.

Oxygen diffusion

Values for the oxygen diffusion rate in 2% agarose gels were estimated from literature (Table S1) [45]. No significant changes in diffusivity were observed for the 318 Da tracer (luciferin) in cell-seeded agarose gels (Fig. 3D), which were assumed to be representative for the changes in diffusivity for the smaller oxygen molecules.

Oxygen consumption rate

Spatiotemporal evolution of oxygen concentration gradients was monitored with OSB. Estimations for the maximal OCR were obtained by fitting simulated radial profiles of oxygen concentration through the experimental data points. Simulated profiles were fitted 12 hours after the initial luciferin bolus, as we measured bioluminescence light emission prior to OSB measurement (see **Materials and Methods** section). Analyses were performed under the assumption of a constant Michaelis-Menten constant, with a value that was adopted from a previous study [5].

Intracellular luciferase concentration

As intracellular luciferase concentrations remained constant throughout the experiments, parameters related to synthesis (*k_s_*) and decay (*k_luc_*) were assumed to be in balance. The total amount of luciferase contained within the hydrogel and determined by the active cell density (see **Materials and Methods** section) was also implemented as a constant during the dynamic bioluminescence emission measurements.

Bioluminescent light generation

The intracellular conversion of luciferin into oxyluciferin followed a Michaelis-Menten kinetics that we modified to describe the influence of available oxygen concentration. Kinetic parameters were quantified under normoxic and hypoxic conditions (Fig. 2C). Parametric changes for intermediate oxygen concentrations were reasoned to follow a square root dependent relationship, similar to ATP [6], another cofactor of the bioluminescence reaction. A second modification to the Michaelis-Menten kinetics was included to account for the influence of macromolecular crowding [21]. With cytoplasmic protein concentrations over 100 g·l^-1^, the intracellular environment can greatly interfere with equilibria and rates of reaction [22,46]. Intracellular crowding resulted in this study in an increased Michaelis-Menten constant compared to the activity in solution. We implemented this change via the introduction of a crowding factor (*Γ*) that had a value of 1.5. This value is however much lower than the intracellular crowding factor of ~7 that has been previously measured for intact 293T cells [23] and a factor of ~2 for firefly luciferase entrapped in 0.2 mol:mol GLTES/DGS sol-gel precursor [47]. Discrepancies were ascribed to the abstraction of reduced luciferin diffusion rates caused by substrate binding or the crowded environment surrounding the enzyme.

Decay initialization time

Exponential decay of bioluminescent light emission was described by a first order kinetic equation. The spatial distribution of cells contained within the hydrogel combined with developed gradients in available luciferin concentration, mandated the introduction of a time initialization parameter (*t_i_*) to accurately describe time- and space-dependent occurrence of bioluminescence reactions and their subsequent activity decay. The criterion for re-initialization of the cytoplasmic bioluminescence reaction was made dependent on the magnitude of intracellular luciferin influx. A transition threshold parameter was introduced to account for the delay in bioluminescence peak activity observed under reduced oxygen concentrations (Fig. 2E).

$$t_{0}^{i+1}= t_{0}^{i}+\left(t-t_{0}^{i}\right)\cdot\frac{\kappa\left(c_{O_{2}}\right)\cdot c_{Dluc,I}^{i+1}\left(z,r,t\right)-c_{Dluc,I}^{i}\left(z,r,t\right)}{\kappa\left(c_{O_{2}}\right)\cdot c_{Dluc,I}^{i+1}\left(z,r,t\right)}$$

for $c_{Dluc,I}^{i+1}\left(z,r,t\right)>c_{Dluc,I}^{i}\left(z,r,t\right)$

$t_{0}^{i+1}= t_{0}^{i}$ for $c_{Dluc,I}^{i+1}\left(z,r,t\right)\leq c_{Dluc,I}^{i}\left(z,r,t\right)$ (**6**)

Initial and boundary conditions

Luciferin substrate was added to the culture medium after 12 h (day 1), 36 h (day 2), 60 h (day 3) cultivation periods. Concentrations were assumed to be homogeneously distributed in the medium. This condition was ensured by initial mixing of the medium at the luciferin bolus time. Initial oxygen concentrations were set to 0.192 mol·m^-3^ (oxygen solubility in water at 37°C) or 21%. Mixing during the luciferin bolus time also resulted in a redistribution of available oxygen concentrations, which we implemented by resetting the oxygen concentration in the cultivation medium to 21%.

The mathematical model was closed by insertion of suitable boundary conditions. No-flux boundary conditions were applied for all boundaries, except for the surface of the cultivation medium where a Dirichlet boundary condition was applied of 21% oxygen concentration during the total experiment time. In axial direction the hydrogel was confined by circular glass plates, preventing oxygen and luciferin diffusion in this direction. No-flux boundary conditions were therefore applied on the glass plate interfaces to prevent this axial diffusion.

**References**

[41] Walsh R, Martin E, Darvesh S (2010) A method to describe enzyme-catalyzed reactions by combining steady state and time course enzyme kinetic parameters. Biochim Biophys Acta 1800: 1-5.

[42] Weiner R, Schmitt BA, Podhaisky H (1997) ROWMAP—a ROW-code with Krylov techniques for large stiff ODEs. Appl Num Math 25: 303-19.

[43] Westrin BA, Axelsson A (1991) Diffusion in gels containing immobilized cells: a critical review. Biotechnol Bioeng 38: 439-46.

[44] Rees DC, Johnson E, Lewinson O (2009) ABC transporters: the power to change. Nat Rev Mol Cell Biol 10: 218-27.

[45] Hulst A, Hens H, Buitelaar R, Tramper J (1989) Determination of the effective diffusion coefficient of oxygen in gel materials in relation to gel concentration. Biotechnol Tech 3: 199-204.

[46] Aoki K, Takahashi K, Kaizu K, Matsuda M (2013) A quantitative model of ERK MAP kinase phosphorylation in crowded media. Sci Rep 3: 1541.

[47] Cruz-Aguado JA, Chen Y, Zhang Z, Elowe NH, Brook MA, et al. (2004) Ultrasensitive ATP detection using firefly luciferase entrapped in sugar-modified sol-gel-derived silica. J Am Chem Soc 126: 6878-9.

[48] Szymanski J, Weiss M (2009) Elucidating the origin of anomalous diffusion in crowded fluids. Phys Rev Lett 103: 038102.

[49] Demol J, Lambrechts D, Geris L, Schrooten J, Van Oosterwyck H (2011) Towards a quantitative understanding of oxygen tension and cell density evolution in fibrin hydrogels. Biomaterials 32: 107-18.
